# Are cortical microcircuits optimized for information flux?—A simulation-based reverse engineering study

**DOI:** 10.3389/fncom.2026.1886601

**Published:** 2026-10-20

**Authors:** Claus Metzner, Ali Ghebleh, Karin Prebeck, Achim Schilling, Andreas Maier, Thomas Kinfe, Patrick Krauss

**Affiliations:** 1 Cognitive Computational Neuroscience Group, Pattern Recognition Lab, Friedrich-Alexander-University Erlangen-Nürnberg (FAU), Erlangen, Germany; 2 Department of Neuromodulation and Neuroprosthetics, University Hospital Mannheim, University Heidelberg, Mannheim, Germany; 3 Neuroscience Lab, University Hospital Erlangen, Erlangen, Germany; 4 BGU Ludwigshafen, Ludwigshafen, Germany

**Keywords:** cortical microcircuits, recurrent neural networks, core-periphery organization, information flux, mutual information, reservoir computing

## Abstract

**Introduction:**

A sufficiently large information flux in recurrent neural networks, quantified by the mutual information between successive network states, has been proposed as a marker of rich information-processing capabilities. This raises the question of whether biological neural networks, such as cortical microcircuits, may be structurally organized to enhance information flux.

**Methods:**

To investigate this possibility, we study a simplified model of the cortical layer 5 architecture, in which a densely and strongly interconnected core population is embedded within a larger supporting network.

**Results:**

Surprisingly, we find that the embedding network exerts a pronounced flux-enhancing effect on the core dynamics. Systematic reverse-engineering analyses reveal that the embedding network primarily generates heterogeneous negative mean inputs that shift the core neurons into a higher-flux operating regime. Replacing the full time-dependent embedding signals by corresponding constant biases preserves this flux-enhancing effect, indicating that the detailed signal dynamics are not essential under the tested conditions. We further show that the information flux can be increased even beyond the biologically embedded case by applying optimized uniform or neuron-specific biases to the core neurons, and that similarly high values can emerge from a simple local adaptive-bias rule. Additional controls show that adaptive regulation and independently tuned inhibitory targeting further improve the embedded network, whereas removing the privileged strong-weight organization of the core abolishes the embedding advantage.

**Discussion:**

These results suggest that the surrounding network acts as a distributed regulator that prevents a strongly recurrent core from becoming trapped in saturated, low-flux states, rather than as an optimizer of information flux. Our findings are relevant both for the functional interpretation of biological neural circuits and for the design of artificial recurrent systems such as reservoir computers.

## 1 Introduction

Biological neural systems must retain, transform, and transmit information while remaining responsive to changing inputs and robust to internal variability (Faisal et al., 2008; Deco et al., 2009). Information theory provides model-independent quantities for describing complementary aspects of these dynamics: entropy characterizes the diversity of accessible states, conditional entropy characterizes the uncertainty of a future state given the present one, and mutual information quantifies the statistical dependence between states or signals (Greenfield and Lecar, 2001). Throughout this work, we use the term *information flux* for the mutual information between successive network states. This measure captures temporal dependence within the network; by itself, however, it neither establishes a causal transfer of information between particular neurons nor guarantees performance on a computational task.

The ability of a neural circuit to sustain such temporally structured dynamics depends not only on its individual neurons but also on how they are connected. Brain networks exhibit non-random, modular, hierarchical, and hub-rich organization across spatial scales (Sporns, 2011; Meunier et al., 2010; Sporns and Betzel, 2016; Van Den Heuvel and Sporns, 2011). In network science, densely interconnected cores or rich clubs embedded in sparser surroundings have been proposed to support a balance between local specialization and global integration (Colizza et al., 2006). At the level of cortical microcircuits, strong and non-random synaptic connections likewise form structured neuronal groups within a more weakly connected background (Song et al., 2005; Perin et al., 2011). These observations motivate the general question of whether core–periphery organization merely reflects anatomical heterogeneity or also regulates the dynamical regime of the core. Importantly, cortical microcircuits are themselves strongly recurrent: neuronal activity is continually fed back through local and longer-range pathways rather than propagating only in one direction.

This contrast has become especially relevant over the past decade, as deep learning has achieved remarkable advances (LeCun et al., 2015; Alzubaidi et al., 2021), driven in part by the rise of large language models (Min et al., 2023). Most contemporary machine-learning systems rely on predominantly feedforward information processing, whereas recurrent neural networks (RNNs) incorporate feedback loops and can operate as autonomous dynamical systems (Maheswaranathan et al., 2019). Once activated, such networks can sustain and transform internal activity even in the absence of ongoing external stimulation.

RNNs possess several fundamental properties that underscore their versatility. They are universal function approximators (Schäfer and Zimmermann, 2006) and can emulate arbitrary dynamical systems (Aguiar et al., 2023). These theoretical capabilities, combined with their practical relevance, have motivated extensive efforts to elucidate their internal working principles. By virtue of their recurrent connectivity, RNNs can accumulate and process information across extended temporal horizons (Jaeger, 2001; Schuecker et al., 2018; Büsing et al., 2010; Dambre et al., 2012; Wallace et al., 2013; Gonon and Ortega, 2021). Moreover, they are capable of forming compact yet expressive state representations, shaped by an interplay between dimensional expansion and compression Farrell et al. (2022).

A substantial body of work has addressed the regulation and stabilization of RNN dynamics, particularly under the influence of intrinsic and extrinsic noise sources (Rajan et al., 2010; Jaeger, 2014; Haviv et al., 2019; Molgedey et al., 1992; Ikemoto et al., 2018; Krauss et al., 2019a; Bönsel et al., 2022; Metzner and Krauss, 2022). Beyond their engineering applications, RNNs have also been studied as models for neural computation in the brain (Barak, 2017). In this context, sparsely connected architectures—resembling biological circuits with limited synaptic connectivity per neuron (Song et al., 2005)—have been shown to support improved memory properties and efficient internal representations (Brunel, 2016; Narang et al., 2017; Gerum et al., 2020; Folli et al., 2018).

Previous studies have shown that recurrent dynamics can be strongly shaped by a small set of structural parameters, including the width of the weight distribution, connection density, and excitation–inhibition balance (Krauss et al., 2019b; Metzner and Krauss, 2022). In particular, a strongly positive balance drives recurrent networks into a fixed-point regime in which weak external signals have little influence, whereas sufficiently strong external perturbations can release the dynamics from the attractor and restore recurrent information flux (Metzner and Krauss, 2022). Work in reservoir computing further indicates that sparsity, heterogeneous connectivity, and other biologically inspired organizational features can alter memory and task performance (Gerum et al., 2020; Metzner et al., 2026). Together, these results motivate a more direct analysis of how a particular cortical connectivity motif changes the temporal dependence of activity within a strongly recurrent subnetwork.

Beyond static structural design, the computational capacity of RNNs can also be modulated dynamically. Counterintuitively, the deliberate injection of external noise into the reservoir may improve performance (Bönsel et al., 2022; Schilling et al., 2022; Krauss et al., 2016, 2019a; Schilling et al., 2021, 2023; Metzner et al., 2024). In regimes where the network becomes trapped in fixed points or low-period attractors, appropriately tuned stochastic perturbations can re-enable dynamical exploration. This mechanism can increase the recurrent information flux Krauss et al. (2019a); Metzner et al. (2024), measured as the mutual information between consecutive global network states, although the relation between this dynamical measure and task-specific computation must be assessed separately. The dependence of information flux on noise intensity can follow a resonance-like profile, termed “Recurrence Resonance”, in which both insufficient and excessive noise reduce temporal dependence.

In our numerical experiments on random signals in recurrent neural networks, we have so far used normally distributed white noise, added independently to the weighted input of each neuron. In a biological setting, such white Gaussian noise could in principle be approximated by the summed spike trains of a large population of statistically independent neurons.

Inputs generated by a finite embedding circuit need not satisfy these simplifying assumptions: they may have neuron-specific means and variances, shared instantaneous covariance, temporal correlations, and non-Gaussian higher-order structure. It is therefore necessary to separate these signal properties experimentally before attributing a dynamical advantage to “complex” biological noise. A surrounding circuit could also regulate the operating point of a recurrent core through approximately constant mean inputs, without implementing an optimizing feedback loop.

Indeed, the organization of cortical microcircuits, in particular the layer 5 architecture reported by Song et al. (2005), exhibits features that appear well suited for such a regulatory function. It includes strongly interconnected excitatory neurons embedded in a broader population of more weakly connected excitatory neurons and inhibitory interneurons.

In this work, we implement a simplified version of this layer 5 architecture, termed the Embedded Core Model (ECM), using probabilistic Boltzmann neurons. The study addresses five concrete questions: (1) Does embedding reproducibly enhance information flux relative to the corresponding isolated core across independently generated networks? (2) Which directed pathways and which statistical components of the embedding input produce that enhancement? (3) Does the effect reflect regulation of the core's operating point rather than architecture-specific optimization? (4) How do changes in successor-state entropy and conditional entropy combine to determine the mutual-information changes? (5) Does the reduced triplet-based indicator track full-state mutual information of the 10-neuron core? To answer these questions, we combine inhibitory-weight and noise sweeps, directional lesions, controlled replacement of embedding signals, numerical and adaptive bias regulation, entropy decomposition, and semianalytical full-state validation. This design distinguishes the question of whether the embedding network *enhances* information flux from the stronger question of whether it *optimizes* it.

## 2 Methods

### 2.1 Embedded core model

To investigate the influence of biologically structured connectivity on network dynamics, we construct a recurrent neural network consisting of *N*_*t*_ = 125 nodes. The model is not intended as a detailed reconstruction of a particular cortical column; rather, it captures selected statistical features of local cortical connectivity, in particular a small set of strong excitatory connections embedded in a larger population of weaker ones (Song et al., 2005).

The network comprises three distinct neuron populations: a subset of *N*_*c*_ = 10 excitatory “core” neurons, a larger population of *N*_*p*_ = 90 excitatory “peripheral” neurons, and a group of *N*_*i*_ = 25 inhibitory “interneurons”. The core neurons are densely and strongly interconnected, whereas the remaining excitatory neurons form a sparsely and weakly connected background network. Interneurons do not connect among themselves but project onto both core and peripheral neurons with negative weights, while receiving excitatory input from both populations.

Connections originating from excitatory neurons are established such that the overall connection density is fixed to *d* = 11.6%, consistent with experimentally measured connection probabilities in cortical layer 5 microcircuits (Song et al., 2005). Synaptic weights *W* for these connections are drawn from a lognormal distribution,


$$\begin{array}{l} p\left(W\right)=\frac{1}{W\sqrt{2\pi \sigma^{2}}}\exp\left(-\frac{{\left(\ln\;W-\mu \right)}^{2}}{2\sigma^{2}}\right), \end{array}$$ (1)

with parameters μ = −0.702 and σ = 0.9355, as reported for synaptic strength distributions in the same study (Song et al., 2005). For each network realization, 30 directed core-to-core edges are sampled without replacement from the 90 possible off-diagonal positions, corresponding to a core connection density of one third. The remaining excitatory edges are sampled without replacement from all allowed non-core positions, giving 1,438 excitatory edges in total. We then draw 1,438 weights from the lognormal distribution, assign the 30 largest values in random order to the selected core-to-core edges, and randomly assign the remaining values to the other selected excitatory edges. Thus, excitatory neurons may target neurons in any of the three populations, whereas inhibitory neurons target only the two excitatory populations. Inhibitory edges are sampled independently at the same density *d*, giving 290 inhibitory edges, and receive the uniform weight *w*_*i*_ = −5 in the reference condition. This inhibitory weight and the other explicitly varied parameters are examined in the parameter sweeps described below. Self-connections are excluded throughout. This construction provides a controlled and reproducible abstraction of the experimentally observed core–periphery organization.

The concentration of strong excitatory weights within the core is a central Song-inspired feature of the ECM. Song et al. (2005) reported a heavy-tailed distribution of synaptic strengths together with a non-random clustering of strong and reciprocal excitatory connections, motivating our representation of a strongly recurrent core embedded in weaker background connectivity. The deterministic assignment of the 30 largest sampled weights to the 30 core-to-core edges is an idealized implementation of this strong-link organization: the precise core size, number of core edges, and weight-ranking rule are modeling choices rather than direct estimates from the experimental data. We therefore tested global random reassignment of excitatory weights and a targeted exchange involving only the 30 core weights (Supplementary Figure S5).

The inhibitory population and its random projections are conventional modeling abstractions rather than features constrained by Song et al., whose measurements concerned excitatory pyramidal-cell connections. The uniform inhibitory weight provides a transparent reference condition. To test whether its equal action on the strongly coupled core and weakly coupled periphery limits the ECM, we additionally varied interneuron-to-core and interneuron-to-periphery strengths independently (Supplementary Figure S3).

The network dynamics is governed by stochastic Boltzmann neurons, which all update synchronously. Given the total synaptic input *u* of a neuron, its on-probability (that is, the probability of producing an output of 1 rather than 0 in the next time step), is given by the logistic activation function


$$\begin{array}{l} p_{1}\left(u\right)=\frac{1}{1+e^{-u/t_{0}}}. \end{array}$$ (2)

The effective temperature is fixed at *t*_0_ = 5 throughout unless stated otherwise. At the beginning of each simulation, every neuron is independently initialized to 1 with probability 1/2. At every subsequent time step, all new binary states are drawn independently conditional on the previous global state and the probabilities given above.

We store the directed weight matrix in the convention *W*_*ij*_ = *W*[target*i*, source*j*]. For neuron *i* in the core network, the total synaptic input *u*_*i*_(*t*) at time step *t* consists of three components from other core neurons, from interneurons and from peripheral neurons:


$$\begin{array}{l} u_{i}\left(t\right)=\underset{k}{\sum }W_{ik}^{CC}z_{k}\left(t\right)+\underset{m}{\sum }W_{im}^{CI}y_{m}\left(t\right)+\underset{n}{\sum }W_{in}^{CP}x_{n}\left(t\right)=\\ =u_{i,cor}\left(t\right)+u_{i,int}\left(t\right)+u_{i,per}\left(t\right)=\\ =u_{i,cor}\left(t\right)+u_{i,emb}\left(t\right), \end{array}$$ (3)

where


$$\begin{array}{l} u_{i,emb}\left(t\right)=u_{i,int}\left(t\right)+u_{i,per}\left(t\right) \end{array}$$ (4)

denotes the total contribution from the embedding networks.

### 2.2 Information flux in the core network

At any given time step *t*, the global state of the core network is defined by a vector **z**(*t*) = (*z*_0_(*t*), *z*_1_(*t*), …), where *z*_*n*_(*t*) ∈ {0, 1} denotes the binary state of neuron *n*. We define the **information flux** within this network as the mutual information *I*(**z**(*t*);**z**(*t* + 1)) between two successive global states. This full-state quantity measures temporal statistical dependence in bits; it is not a directed or causal measure of information transfer between individual neurons.

For the numerical evaluation of the mutual information


$$\begin{array}{l} I\left(X;Y\right)=\underset{x}{\sum }\underset{y}{\sum }P\left(x,y\right)\log_{2}\left(\frac{P\left(x,y\right)}{P\left(x\right)P\left(y\right)}\right), \end{array}$$ (5)

we simulate long time series of network states and estimate the joint probability distribution *P*(*x, y*) by counting pairs of successive states (*x* = **z**(*t*), *y* = **z**(*t* + 1)), excluding all terms for which one or more of the involved probabilities are zero. All empirical information quantities reported below use this plug-in estimator and logarithms to base two.

For the complete 10-neuron core, the joint distribution of successive states contains 2^10^ × 2^10^ entries. Direct empirical estimation across all ensemble members and experimental conditions would therefore require substantially longer trajectories than estimation for small neuronal groups and would be especially sensitive to sparsely sampled state pairs. We consequently reserve the full-state calculation for the semianalytical validation described in Section 2.9, where the autonomous isolated core permits explicit construction of its Markov transition matrix. For the repeated simulations, we use the lower-dimensional indicator defined next.

We select three disjoint triplets within the core network before performing the analyses: *A* = {0, 1, 2}, *B* = {3, 4, 5}, and *C* = {6, 7, 8}. Neuron 9 is not included, because three equally sized, non-overlapping triplets contain nine of the ten core neurons. The same grouping is held fixed across all network realizations and experimental conditions. We then define the **intra-triplet flux** *I*_*intra*_ by computing *I*_*AA*_ = *I*(**z**_*A*_(*t*);**z**_*A*_(*t* + 1)), *I*_*BB*_, and *I*_*CC*_ independently for each triplet and averaging over the three values. In addition, we define the **inter-triplet flux** *I*_*inter*_ by computing *I*_*AB*_ = *I*(**z**_*A*_(*t*);**z**_*B*_(*t* + 1)), as well as *I*_*AC*_, *I*_*BC*_, *I*_*BA*_, *I*_*CA*_, and *I*_*CB*_, and averaging over these six ordered pairwise values. Both averages are expressed in bits. We report their scaled sum as the triplet-based information-flux indicator


$$\begin{array}{l} f=10^{3}\left(I_{intra}+I_{inter}\right). \end{array}$$ (6)

Thus, one indicator unit corresponds to 10^−3^ bit of the summed triplet measure. The scaling changes only numerical presentation.

To distinguish changes in state diversity from changes in unpredictability, we additionally use the equivalent identity


$$\begin{array}{l} I\left(X;Y\right)=H\left(Y\right)-H\left(Y|X\right), \end{array}$$ (7)

where


$$\begin{array}{l} H\left(Y\right)=-\underset{y}{\sum }P\left(y\right)\log_{2}P\left(y\right), \end{array}$$ (8)

$$\begin{array}{l} H\left(Y|X\right)=-\underset{x}{\sum }\underset{y}{\sum }P\left(x,y\right)\log_{2}P\left(y|x\right). \end{array}$$ (9)

For the intra-triplet quantities, *H*_*intra*_ and *H*_*cond,intra*_ denote the averages of *H*(*Y*) and *H*(*Y*|*X*) over *AA*, *BB*, and *CC*. The corresponding inter-triplet quantities *H*_*inter*_ and *H*_*cond,inter*_ are averaged over the six ordered cross-triplet pairs. By construction, *I*_*intra*_ = *H*_*intra*_ − *H*_*cond,intra*_ and *I*_*inter*_ = *H*_*inter*_ − *H*_*cond,inter*_.

The indicator *f* is a deliberately compressed summary of temporal dependence among the selected triplets. It is not assumed to equal the full 10-neuron mutual information, and its absolute magnitude should not be interpreted as a full-state information rate. We therefore use it primarily for paired comparisons across conditions. Its agreement with full-state mutual information, its ability to track parameter-induced changes, and its robustness to alternative triplet partitions are evaluated separately in Section 2.9.

For the descriptive analysis of statistical dependencies throughout the complete 125-neuron ECM, we additionally calculated pairwise mutual information between individual binary neurons. For an ordered neuron pair (*i, j*) and lag Δ*t*, this quantity was defined as


$$\begin{array}{l} I_{ij}^{\left(\Delta t\right)}=I\left(z_{i}\left(t\right);z_{j}\left(t+\Delta t\right)\right), \end{array}$$ (10)

using the plug-in estimator in Equation 5. We considered Δ*t* = 0, which measures symmetric simultaneous dependence, and Δ*t* = 1, for which row *i* and column *j* describe temporal dependence from source neuron *i* to target neuron *j* one update later. The latter remains a statistical association and is not interpreted as a causal information-transfer measure. Full 125 × 125 matrices were retained for visualization. To obtain a population-level matrix, we averaged $I_{ij}^{\left(\Delta t\right)}$ over all ordered pairs with *i* in a given source population and *j* in a given target population. For entries in which source and target were the same population, self-pairs *i* = *j* were excluded at both lags. This prevents the simultaneous identity $I_{ii}^{\left(0\right)}=H\left(z_{i}\right)$, together with the unequal population sizes, from artificially inflating comparisons of within-population dependence. Pairwise quantities were calculated from 50,000 recorded transitions in two independent trajectories, averaged within each network, and then summarized across the 25 network realizations as described in Section 2.3.

### 2.3 Ensemble design and statistical analysis

To distinguish effects of embedding from effects specific to a single randomly drawn network, we generated an ensemble of 25 independent ECM weight matrices (network identifiers 000–024). Each matrix was generated once, stored together with its generation metadata and checksum, and then reused in every condition applied to that network. Thus, comparisons between embedded and isolated cores, parameter values, lesions, and replacement inputs were paired at the level of the core weight matrix. No generated network was excluded.

The independent network realization, not an individual simulation trajectory, was the primary statistical unit. Unless stated otherwise, a trajectory comprised 5,000 burn-in updates followed by 50,000 recorded state transitions. Where a condition was simulated more than once, its metric was first averaged across those repeats within each network. This produced one value per network and condition and prevented repeated trajectories from being counted as independent network samples. Conditions intended for direct comparison used the same network and corresponding simulation repeats. The shorter trajectories used only during numerical bias optimization are described in the corresponding subsection.

Ensemble summaries are reported as arithmetic means across networks. For a comparison of conditions *a* and *b*, we first calculated the within-network contrast $d_{i}={\bar{x}}_{i,a}-{\bar{x}}_{i,b}$ and then summarized the distribution of *d*_*i*_ across the 25 networks. Ninety-five-percent confidence intervals for ensemble means were obtained by nonparametric percentile bootstrap: network identifiers were sampled with replacement 20,000 times, all paired observations belonging to a sampled network were kept together, and the 2.5th and 97.5th percentiles of the bootstrap means were used as interval limits. For parameter sweeps, this procedure was applied separately at each parameter value; for lesions and replacement controls, it was applied to the paired within-network contrasts. No *p*-value threshold was used, and the confidence intervals quantify variation across independently generated networks rather than uncertainty due solely to finite trajectory length.

Additional robustness analyses extended this paired ensemble design to adaptive regulation in the complete ECM, independent variation of inhibitory projections to the core and periphery, input-resolved lesion effects, overlap of embedding projections, activity changes during the noise sweep, alternative assignments of the strong core weights, and alternative confidence-interval and hypothesis-test procedures. The corresponding protocols and complete numerical results are provided in the Supplementary Methods and Supplementary Figures S2–S6.

### 2.4 Parameter sweeps and lesion experiments

We performed two parameter sweeps for every network realization. First, in the complete embedded network, we varied the common weight of all inhibitory projections over the values


$$w_{i}\in \left\{-10,-8,-6,-5,-4,-2,0\right\},$$

while keeping *t*_0_ = 5 and all excitatory weights fixed. This sweep includes the reference value *w*_*i*_ = −5. Second, we isolated the 10-neuron core and added independent Gaussian noise to the scaled activation argument of every core neuron,


$$\begin{array}{l} p_{i}\left(t+1\right)=\frac{1}{1+\exp\left[-\left(u_{i}\left(t\right)/t_{0}+\eta_{i}\left(t\right)\right)\right]},\;\eta_{i}\left(t\right)\sim N\left(0,\sigma^{2}\right). \end{array}$$ (11)

The noise samples were independent across neurons and time steps. We tested


σ∈{0,0.5,1,1.5,2,2.5,3,4,6,8,10},

where σ = 0 is the noise-free isolated-core reference. Both sweeps used 5,000 burn-in updates and 50,000 recorded transitions at every point, with the same stored weight matrix retained across all points belonging to a network. For each point, we measured *I*_*intra*_, *I*_*inter*_, the indicator *f*, their entropy and conditional-entropy components, and the mean on-probability of the core neurons. Any reported optimum denotes the maximum among the tested discrete parameter values; no continuous interpolation or network-specific retuning was applied.

For the lesion experiments, we used the complete embedded network at *t*_0_ = 5 and *w*_*i*_ = −5 without additional noise. A lesion was implemented by setting all weights in one selected directed population-to-population block to zero while leaving the rest of the same network realization unchanged. In addition to the intact reference, we tested seven lesions: core-to-periphery, core-to-interneurons, interneurons-to-core, periphery-to-core, periphery-to-interneurons, interneurons-to-periphery, and the combined core-to-embedding lesion, which simultaneously removed the core-to-periphery and core-to-interneuron projections. Direction names refer to signal flow from source to target. Each condition used the standard 5,000-update burn-in and 50,000-transition measurement period. Lesion effects were defined as the within-network difference between the lesioned and intact conditions and were summarized across networks as described in Section 2.3.

### 2.5 Embedding-signal replacements and gaussian controls

To separate the effects of the mean, marginal variance, instantaneous cross-neuron covariance, and temporal structure of the embedding input, we first simulated the complete ECM and recorded, for every core neuron, the input *u*_*i,emb*_(*t*) defined in Equation 4. For each network realization, this reference simulation used a 5,000-update burn-in and 50,000 recorded transitions. All target statistics were estimated from the post-burn-in part of the resulting 10-dimensional input time series. In particular, we calculated the neuron-specific mean vector **μ**, marginal standard deviations **σ**, and instantaneous spatial covariance matrix **Σ**, with


$$\begin{array}{l} \mu_{i}={\langle u_{i,emb}\left(t\right)\rangle }_{t},\;\Sigma_{ij}={\langle \left(u_{i,emb}\left(t\right)-\mu_{i}\right)\left(u_{j,emb}\left(t\right)-\mu_{j}\right)\rangle }_{t},\\ \sigma_{i}=\sqrt{\Sigma_{ii}}. \end{array}$$ (12)

We then retained the original recurrent core-to-core weight matrix but replaced its embedding input by a prescribed external signal *s*_*i*_(*t*). The update probability in these replacement simulations was


$$\begin{array}{l} p_{i}\left(t+1\right)=\frac{1}{1+\exp\left[-\left(u_{i,cor}\left(t\right)+s_{i}\left(t\right)\right)/t_{0}\right]}. \end{array}$$ (13)

Six conditions were evaluated for every network. In *full embedding*, the core remained embedded in the dynamically coupled ECM and therefore received the original *u*_*i,emb*_(*t*). In *isolated core*, *s*_*i*_(*t*) = 0. In *empirical input replay*, the recorded vector time series was replayed exactly, *s*_*i*_(*t*) = *u*_*i,emb*_(*t*), while the surrounding populations were absent; this preserves the realized input sequence but removes any ongoing feedback between the replay-driven core and the embedding network. In *per-neuron matched mean*, each core neuron received its own constant empirical mean, *s*_*i*_(*t*) = μ_*i*_. This condition is not optimized and should not be confused with the per-neuron optimized biases described in Section 2.6.

The remaining two conditions used temporally white Gaussian signals that retained different subsets of the measured input statistics. For *independent Gaussian (matched* μ_*i*_, σ_*i*_*)*, each neuron received independent samples $s_{i}\left(t\right)\sim N\left(\mu_{i},\sigma_{i}^{2}\right)$, with independence across neurons and time. The post-burn-in samples were recentered and rescaled column by column so that their finite-sample means and standard deviations matched μ_*i*_ and σ_*i*_. For *spatial-covariance-matched Gaussian (matched* **μ**, **Σ***)*, the signal vectors were sampled from N(μ,Σ). A finite Gaussian sample was centered, whitened using an eigendecomposition of its sample covariance, recolored with the symmetric square root of **Σ**, and shifted by **μ**. Consequently, this control includes the neuron-specific mean biases and reproduces the complete instantaneous spatial covariance matrix over the measurement interval.

All replacement-core simulations used the same standard burn-in and measurement lengths and the same stored core matrix as their full-embedding reference. Gaussian signals were generated independently for each network and condition. We verified every generated signal by recording the root-mean-square errors of its mean and marginal standard deviation relative to the empirical input, together with the relative Frobenius errors of its instantaneous and lag-one covariance matrices. Neither Gaussian control was designed to reproduce temporal covariance. Therefore, a difference between the empirical embedding input and the spatial-covariance-matched Gaussian control cannot by itself be attributed uniquely to non-Gaussian marginal distributions; it may also reflect temporal correlations or higher-order dependencies. Paired effects relative to full embedding were summarized across networks according to Section 2.3.

### 2.6 Numerical optimization of core biases

Bias optimization was performed separately for the isolated core of every network realization. A constant bias vector **b** = (*b*_0_, …, *b*_9_) was supplied as the external signal in Equation 13, *s*_*i*_(*t*) = *b*_*i*_, and the triplet-based indicator *f* was used as the objective. Candidate selection used shorter trajectories consisting of 1,000 burn-in updates and 10,000 recorded transitions. Each candidate objective was averaged over two independent simulations. The temperature remained *t*_0_ = 5.

For the *optimized uniform bias*, all ten components were constrained to a common value, *b*_*i*_ = *b*. We first evaluated a coarse grid from *b* = −15 to *b* = 2 in increments of 0.5. Around the best coarse-grid value, we then evaluated a second grid extending 0.5 in either direction in increments of 0.1. The tested value with the largest average *f* was retained for that network. Thus, the uniform optimum was selected from an explicitly sampled grid and did not rely on interpolation.

For the *per-neuron optimized biases*, we used an elitist evolutionary search in the 10-dimensional interval −20 ≤ *b*_*i*_ ≤ 5. Four independent starting vectors were examined for each network: the optimized uniform-bias vector, the per-neuron matched empirical mean **μ** from Section 2.5, and two random vectors whose components were drawn uniformly from [−12, −3]. Starting vectors were clipped to the allowed interval. From a parent vector **b**^(*k*)^, each iteration generated ten offspring according to


$$\begin{array}{l} {\text{b}}^{\prime }=\mathrm{clip}\left({\text{b}}^{\left(k\right)}+\epsilon ,-20,5\right),\;\epsilon_{i}\sim N\left(0,s_{k}^{2}\right). \end{array}$$ (14)

The parent itself was retained as an eleventh candidate, and the candidate with the largest training objective became the next parent. The mutation scale started at *s*_0_ = 2, was multiplied by 0.85 after each iteration, and was bounded below by 0.2. Each of the four starts was evolved for a fixed 12 iterations, and the final vector with the largest training objective across starts was retained. Elitism made the best recorded objective non-decreasing within each search. We stored the complete search traces to assess stabilization, but used neither early stopping nor a claim of guaranteed global convergence; “optimized” therefore denotes the best solution found under this fixed search budget.

To evaluate the selected biases independently of the search trajectories, we used two independent held-out simulations, neither of which entered candidate selection. For each simulation and network, we used 5,000 burn-in updates followed by 50,000 recorded transitions under five conditions: the full embedded ECM, the isolated core without external input, the per-neuron matched mean **μ**, the optimized uniform bias, and the per-neuron optimized bias vector. All bias conditions used the same stored recurrent core matrix. We recorded *I*_*intra*_, *I*_*inter*_, *f*, and their entropy and conditional-entropy components. The two held-out estimates were averaged within each network before the paired ensemble statistics described in Section 2.3 were calculated.

### 2.7 Mutual information of successive activations in a self-coupled noisy Boltzmann neuron

We consider a single binary Boltzmann neuron with states


$$\begin{array}{l} x_{t}\in \left\{0,1\right\}, \end{array}$$ (15)

recurrent self-coupling strength *w*, bias *b*, and additive noise ξ_*t*_ drawn independently at each time step from a symmetric uniform distribution on [−*a, a*]. The total input determining *x*_*t*+1_ is therefore


$$\begin{array}{l} u_{t}=wx_{t}+b+\xi_{t}. \end{array}$$ (16)

$$\begin{array}{l} p_{1}\left(u\right)=\frac{1}{1+\exp\left(-u\right)}. \end{array}$$ (17)

Here, *w*, *b*, and *a* are expressed in units of the effective temperature, so that this formulation is equivalent to Equation 2 after division of all inputs by *t*_0_.

Averaging over the uniform noise yields the effective activation probability


$$\begin{array}{l} F(u;a)=\frac{1}{2a}\int_{-a}^{a}p_{1}(u+\xi )d\xi =\frac{1}{2a}[\ln\left(1+\exp(u+a)\right)\\ \;-\ln\left(1+\exp(u-a)\right)]. \end{array}$$ (18)

For the noise-free case, the continuous limit *F*(*u*; 0) = *p*_1_(*u*) is used. For the two possible previous states, we define


$$\begin{array}{l} A\equiv F\left(b;a\right),\;C\equiv F\left(w+b;a\right). \end{array}$$ (19)

Hence the transition probabilities are


$$\begin{array}{ll} P\left(1|0\right)=A, & P\left(0|0\right)1-A, \end{array}$$ (20)

$$\begin{array}{ll} P\left(1|1\right)=C, & P\left(0|1\right)1-C, \end{array}$$ (21)

The corresponding Markov transition matrix is


$$\begin{array}{l} T=\left(\begin{array}{cc} 1-A & A\\ 1-C & C \end{array}\right). \end{array}$$ (22)

Let π_0_ and π_1_ denote the stationary probabilities of states 0 and 1. From


$$\begin{array}{l} \pi_{1}=\pi_{0}A+\pi_{1}C,\;\pi_{0}+\pi_{1}=1, \end{array}$$ (23)

one obtains


$$\begin{array}{l} \pi_{1}=\frac{A}{1-C+A},\;\pi_{0}=\frac{1-C}{1-C+A}. \end{array}$$ (24)

$$\begin{array}{l} p_{ij}\equiv P\left(x_{t}=i,x_{t+1}=j\right), \end{array}$$ (25)

follow as


$$\begin{array}{l} p_{00}=\pi_{0}\left(1-A\right)=\frac{\left(1-C\right)\left(1-A\right)}{1-C+A}, \end{array}$$ (26)

$$\begin{array}{l} p_{01}=\pi_{0}A=\frac{A\left(1-C\right)}{1-C+A}, \end{array}$$ (27)

$$\begin{array}{l} p_{10}=\pi_{1}\left(1-C\right)=\frac{A\left(1-C\right)}{1-C+A}, \end{array}$$ (28)

$$\begin{array}{l} p_{11}=\pi_{1}C=\frac{AC}{1-C+A}. \end{array}$$ (29)

The mutual information between two successive activations is


$$\begin{array}{l} I\left(x_{t};x_{t+1}\right)=\underset{i,j\in \left\{0,1\right\}}{\sum }p_{ij}\log_{2}\left(\frac{p_{ij}}{\pi_{i}\pi_{j}}\right). \end{array}$$ (30)

This can be rewritten in terms of entropy and conditional entropy as


$$\begin{array}{l} I\left(x_{t};x_{t+1}\right)=H\left(x_{t+1}\right)-H\left(x_{t+1}|x_{t}\right), \end{array}$$ (31)

where


$$\begin{array}{l} H\left(x_{t+1}\right)=-\underset{j\in \left\{0,1\right\}}{\sum }\pi_{j}\log_{2}\pi_{j}, \end{array}$$ (32)

and


$$\begin{array}{l} H\left(x_{t+1}|x_{t}\right)=-\underset{i,j\in \left\{0,1\right\}}{\sum }p_{ij}\log_{2}P\left(x_{t+1}=j|x_{t}=i\right). \end{array}$$ (33)

Introducing the binary entropy function


$$\begin{array}{l} h_{2}\left(p\right)=-p\log_{2}p-\left(1-p\right)\log_{2}\left(1-p\right), \end{array}$$ (34)

these quantities become


$$\begin{array}{l} H\left(x_{t+1}\right)=h_{2}\left(\pi_{1}\right), \end{array}$$ (35)

and


$$\begin{array}{l} H\left(x_{t+1}|x_{t}\right)=\pi_{0}h_{2}\left(A\right)+\pi_{1}h_{2}\left(C\right). \end{array}$$ (36)

$$\begin{array}{l} I\left(x_{t};x_{t+1}\right)=h_{2}\left(\pi_{1}\right)-\pi_{0}h_{2}\left(A\right)-\pi_{1}h_{2}\left(C\right). \end{array}$$ (37)

Substituting the stationary probabilities finally yields


$$\begin{array}{l} I\left(x_{t};x_{t+1}\right)=h_{2}\left(\frac{A}{1-C+A}\right)-\frac{\left(1-C\right)h_{2}\left(A\right)+Ah_{2}\left(C\right)}{1-C+A}, \end{array}$$ (38)

with


$$\begin{array}{l} A=F\left(b;a\right),\;C=F\left(w+b;a\right), \end{array}$$ (39)

and


$$\begin{array}{l} F\left(u;a\right)=\frac{1}{2a}\left[\ln\left(1+\exp\left(u+a\right)\right)-\ln\left(1+\exp\left(u-a\right)\right)\right]. \end{array}$$ (40)

Thus, the mutual information is obtained explicitly as a function of the self-coupling strength *w*, the bias *b*, and the half-width *a* of the added uniform noise.

### 2.8 Neurons with adaptive bias

The output entropy of a binary Boltzmann neuron is maximal when the on-probability is *p*_1_ = 1/2. In this case, the time-averaged output of the neuron satisfies 〈_*z*_*i*_(*t*)〉*t*_ = 1/2. To drive the neuron toward this maximum-entropy operating point, we let the bias *b*_*i*_(*t*) adapt dynamically according to


$$\begin{array}{l} b_{i}\left(t\right)=b_{i}\left(t-1\right)-\epsilon \left(z_{i}\left(t\right)-\frac{1}{2}\right), \end{array}$$ (41)

where ϵ = 0.01 is a small adaptation rate constant. This update rule implements a local negative feedback mechanism: if the neuron is active more often than desired (*z*_*i*_(*t*) > 1/2), the bias is decreased, whereas for too infrequent activation (*z*_*i*_(*t*) < 1/2), the bias is increased. Over time, this drives the average activity toward 1/2. This target maximizes the marginal output entropy of each binary neuron, but it does not by itself guarantee maximal mutual information between successive network states, because the latter also depends on conditional entropy.

We tested the adaptive rule separately for every isolated core in the ensemble at *t*_0_ = 5, without embedding input or additional noise. All ten biases were initialized to zero and adapted for 100,000 synchronous updates. Biases and mean activities were recorded in blocks of 100 updates. Two independent adaptation trajectories were run for each network. For each trajectory, the learned neuron-specific bias vector was defined as the component-wise mean of the recorded biases over the final 20,000 adaptation steps. The corresponding mean activities were retained to verify convergence toward the target probability.

For an independent assessment of the resulting operating point, adaptation was then stopped and each learned bias vector was held constant. It was evaluated in two independent simulations, each with 5,000 burn-in updates and 50,000 recorded transitions. We measured *I*_*intra*_, *I*_*inter*_, *f*, and their entropy and conditional-entropy components. The four estimates arising from two adaptation trajectories and two evaluation simulations were averaged within each network before comparison with the other conditions and ensemble analysis according to Section 2.3.

### 2.9 Full-state mutual information and validation metrics

We validated the triplet-based indicator against the full-state mutual information of the complete 10-neuron core for a prespecified subset of five network realizations (identifiers 000–004). The validation used the autonomous isolated core at *t*_0_ = 5 with a constant uniform bias *b* and no additional noise. Because this system is a finite, synchronously updated Markov chain, its complete transition matrix can be constructed directly rather than estimated from a long trajectory.

For every predecessor state **x** ∈ {0, 1}^10^, the conditional on-probability of neuron *j* was


$$\begin{array}{l} q_{j}\left(\text{x};b\right)={\left[1+\exp\left(-\frac{\sum_{k}W_{jk}^{CC}x_{k}+b}{t_{0}}\right)\right]}^{-1}. \end{array}$$ (42)

Conditional independence of the Boltzmann draws then gives the probability of every successor state **y** ∈ {0, 1}^10^ as


$$\begin{array}{l} T_{\text{x}\text{y}}=P\left(\text{y}|\text{x}\right)=\overset{9}{\underset{j=0}{\prod }}q_{j}{\left(\text{x};b\right)}^{y_{j}}{\left[1-q_{j}\left(\text{x};b\right)\right]}^{1-y_{j}}. \end{array}$$ (43)

This yields a 1024 × 1024 row-stochastic transition matrix. Starting from the uniform distribution over states, we iterated **π**^(*n*+1)^ = **π**^(*n*)^*T* until the maximum absolute change of any state probability was below 10^−13^, allowing at most 100,000 iterations. The stationary joint distribution of successive states was then


$$\begin{array}{l} P\left(\text{x},\text{y}\right)=\pi_{\text{x}}T_{\text{x}\text{y}}. \end{array}$$ (44)

From this distribution, we calculated the full-state mutual information using Equation 5, together with the full successor-state entropy and conditional entropy from Equation 7. The method is termed semianalytical because all transition probabilities are evaluated analytically whereas the stationary distribution is obtained by numerical iteration; the resulting information quantities are exact up to the stated convergence tolerance.

For each network, we evaluated uniform biases from *b* = −15 to *b* = 2 in increments of 0.5 and additionally included that network's previously selected optimized uniform bias from Section 2.6. This produced 36 bias points per network and 180 full-state joint distributions in total. For every joint distribution, the fixed triplet quantities *I*_*intra*_, *I*_*inter*_, and *f* were obtained by exact marginalization onto the original groups *A*, *B*, and *C* defined in Section 2.2. Thus, the full-state and triplet quantities compared in this validation were derived from the same joint distribution and were not affected by finite-sample differences. At bias values also present in the simulation-based uniform-bias search, we additionally compared the exact triplet indicator with its empirical estimate.

Agreement between full-state mutual information and the triplet indicator was quantified separately within each network by Spearman's rank correlation across bias values. We also calculated the fraction of successive bias intervals for which both measures had the same sign of change and the absolute difference between the sampled bias maximizing full-state mutual information and that maximizing *f*. For the exact-vs.-empirical triplet comparison, we calculated the pooled Spearman correlation and the mean absolute indicator error over the available matched points. These analyses assess whether the indicator tracks changes and preserves the location of an optimum; because *f* combines lower-dimensional marginal quantities, its absolute value is not expected to equal the full-state mutual information.

To test sensitivity to the arbitrary neuron grouping, we repeated the exact marginalization for 30 alternative partitions in addition to the original partition. The alternatives were generated once and then held fixed for all analyses. For each possible omitted neuron, three partitions were sampled by randomly dividing the remaining nine neurons into three disjoint triplets, yielding balanced coverage of all ten choices of omitted neuron. Every partition was evaluated for all 180 network–bias joint distributions, giving 5,580 partition-specific points. Spearman correlation, sign-of-change agreement, and the absolute error in the maximizing bias were then calculated for each network and partition.

### 2.10 Use of generative artificial intelligence

OpenAI Codex (GPT-5, OpenAI, San Francisco, CA, United States; https://openai.com/) was used to assist with code development, debugging, and documentation. All AI-assisted code was reviewed and tested by the authors, and all reported analyses were executed and validated by the authors.

## 3 Results

### 3.1 Optimizing the network's operating point

Our full Embedded Core Model (ECM), described in Section 2.1 and illustrated schematically in Figure 1a, consists of three interconnected populations: a core network of *N*_*c*_ = 10 neurons, a periphery network of *N*_*p*_ = 90 neurons, and a group of *N*_*i*_ = 25 interneurons. The peripheral and interneuron populations together form the embedding network of the core. Representative connection-weight matrices of the core and the complete system are shown in Figures 1b, c.

**Figure 1:**
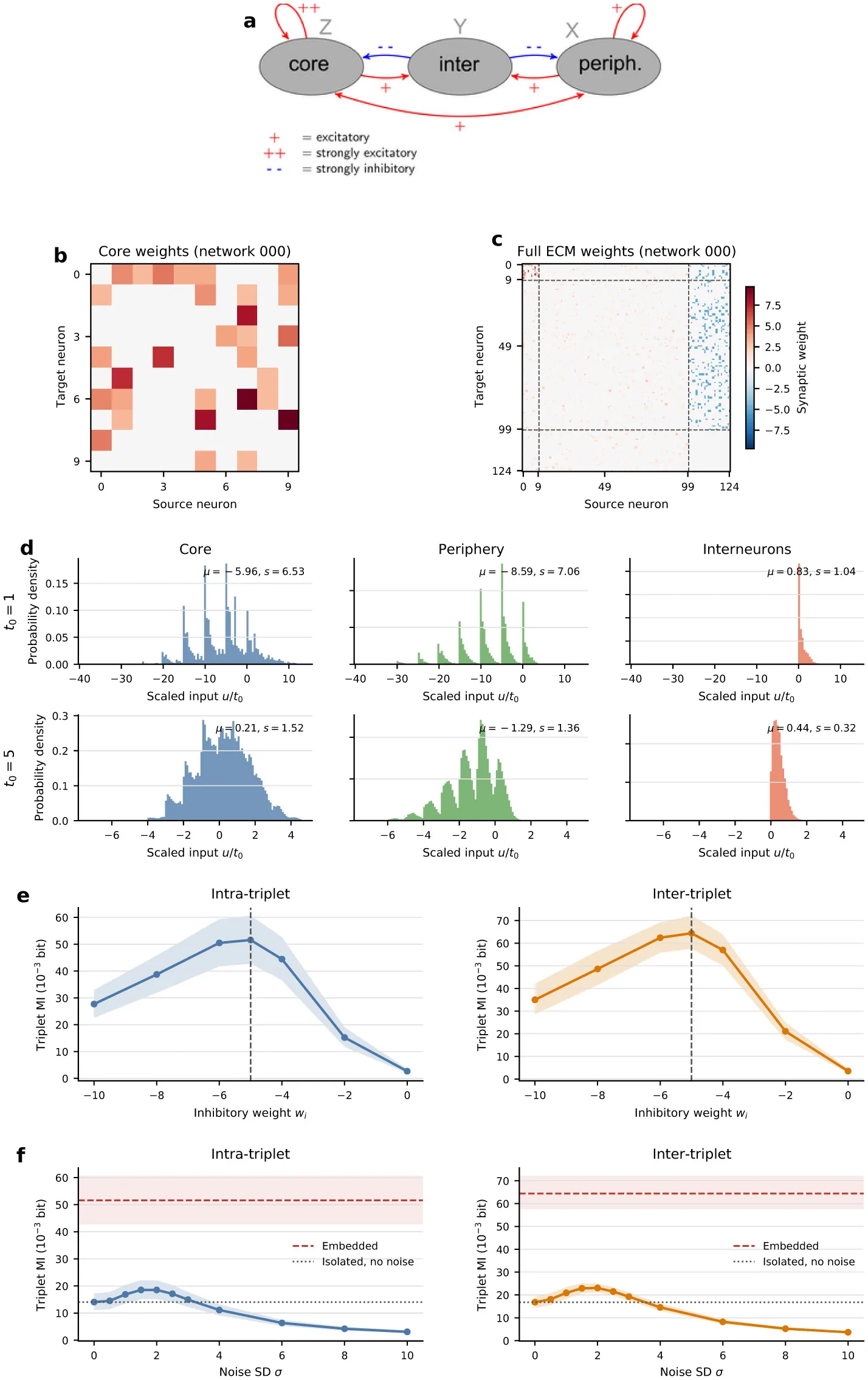
The Embedded Core Model and its operating regime. **(a)** Population-level organization of the excitatory core, excitatory periphery, and inhibitory interneurons. **(b, c)** Core and complete connection-weight matrices for representative network realization 000; dashed lines in **(c)** delimit the three populations. Matrix entries follow the convention *W*[target, source]. **(d)** Distributions of the scaled total synaptic input *u*/*t*_0_ in the three populations of network 000 at *t*_0_ = 1 and *t*_0_ = 5 (5,000 burn-in updates and 50,000 recorded updates; dynamics seed 10,001). **(e)** Intra- and inter-triplet mutual information in the embedded core as functions of the common inhibitory weight. **(f)** Corresponding quantities in the isolated core as functions of the standard deviation of additional Gaussian noise. Lines and shaded bands in **(e, f)** show ensemble means and bootstrap 95% confidence intervals across 25 independently generated networks. The vertical dashed line in **(e)** marks the reference value *w*_*i*_ = −5. In **(f)**, the red dashed line and band denote the embedded-core reference at *w*_*i*_ = −5, and the gray dotted line denotes the isolated core without added noise.

The connectivity pattern and the log-normal distribution of excitatory weights reflect measured properties of cortical microcircuits (Song et al., 2005). However, the effective scale of the synaptic inputs and the relative strength of inhibition are not sufficiently constrained by these data. We therefore first determined a suitable operating regime before comparing the embedded and isolated cores.

For the unscaled network, corresponding to *t*_0_ = 1, the distributions of the scaled input *u*/*t*_0_ extended far into the saturated range of the Boltzmann response in all three populations (Figure 1d, upper row). Increasing the temperature to *t*_0_ = 5 compressed these distributions toward the responsive part of the activation function (Figure 1d, lower row). We therefore retained *t*_0_ = 5 throughout the following analyses.

We next varied the common inhibitory weight *w*_*i*_ across all 25 independently generated networks. Both the intra-triplet and inter-triplet mutual information showed a broad, non-monotonic dependence on inhibitory strength, with their ensemble means reaching the largest sampled values at *w*_*i*_ = −5 (Figure 1e). At this point, the mean intra-triplet contribution was 0.052 bit (95% confidence interval: 0.043–0.061 bit), and the mean inter-triplet contribution was 0.064 bit (0.058–0.072 bit), corresponding to a mean triplet-based flux indicator of *f* ≈ 116. Weaker inhibition moved the networks toward strongly excited, low-flux dynamics, whereas substantially stronger inhibition also reduced both contributions.

Thus, the reference parameters *t*_0_ = 5 and *w*_*i*_ = −5 place the ECM near the maximum of the tested inhibitory-weight range, and this operating point is reproduced at the ensemble level rather than being a peculiarity of a single network realization. We use it as the embedded reference condition below. This parameter choice establishes a consistent high-flux regime for the comparisons that follow; it does not by itself imply that the embedding architecture globally optimizes information flux.

### 3.2 Isolated core network

We next removed the periphery and interneuron populations and analyzed each core network in complete isolation. Information flux then decreased sharply in every one of the 25 paired network realizations (Figures 1e, f). Across the ensemble, the mean intra-triplet contribution fell from 0.052 bit in the embedded condition to 0.014 bit in the isolated core (95% confidence interval: 0.011–0.017 bit), and the inter-triplet contribution fell from 0.064 bit to 0.017 bit (0.014–0.019 bit). Accordingly, the mean flux indicator decreased from approximately 116 to 30.9.

This loss of information flux reflects the strong and purely excitatory recurrent coupling within the core. Without regulating input from the embedding populations, positive feedback drove the isolated networks toward persistently high activity: the ensemble-mean on-probability was 0.881 (95% confidence interval: 0.873–0.891), compared with 0.548 in the embedded reference condition. The dynamics therefore spent much of their time in a restricted subset of states in which most core neurons were active.

Mutual information between successive states requires both sufficiently diverse state occupation and reproducible temporal dependence. The isolated core strongly violates the first condition: confinement near the highly active state reduces the accessible state-space diversity and thereby limits the information that one state can carry about the next. The embedding network mitigates this trapping and reproducibly moves the core into a higher-flux regime.

In principle, this improvement could arise from structured feedback between the core and the surrounding populations, from a shift of the mean operating point, or from fluctuating input with beneficial statistical properties. We distinguish these possibilities in the analyses below, beginning with the simpler question of whether independent Gaussian noise can rescue information flux in the isolated core.

### 3.3 Noise-enhanced information flux

As established in earlier work (Krauss et al., 2019a; Metzner et al., 2024), appropriately tuned noise can increase information flux when recurrent networks are confined to a restricted region of state space. We therefore added independent white Gaussian noise to every neuron of each isolated core and varied its standard deviation σ (Figure 1f). The ensemble means displayed a resonance-like dependence on noise strength: both triplet contributions initially increased, reached their largest or nearly largest sampled values around σ = 2, and declined again at higher noise levels. This characteristic behavior is referred to as Recurrence Resonance.

At σ = 2, the mean intra-triplet contribution was 0.0185 bit (95% confidence interval: 0.0151–0.0220 bit), and the mean inter-triplet contribution was 0.0231 bit (0.0212–0.0250 bit). The corresponding mean flux indicator was *f* = 41.6, compared with *f* = 30.9 without added noise. Thus, independent Gaussian noise produced a moderate rescue of information flux at the ensemble level. The increase was not universal at this fixed noise amplitude, however: 22 of the 25 isolated cores showed a higher indicator at σ = 2 than at σ = 0.

This partial rescue coincided with movement away from the saturated operating point. The ensemble-mean core activity decreased from 0.8815 without noise to 0.7731 at σ = 2, a paired change of −0.1084 (95% confidence interval: −0.1134 to −0.1034) that occurred in all 25 networks (Supplementary Figures S5a, b). At still larger noise amplitudes, increasing transition uncertainty outweighed the benefit of broader state occupation.

Crucially, even the maximum of the ensemble-mean noise curve remained far below the embedded reference value of approximately 116, and the embedded condition outperformed the isolated core at σ = 2 in every paired network realization. Stronger noise ultimately became detrimental; at σ = 10, the mean indicator had fallen to 6.76, below the noise-free isolated-core value. Independent Gaussian fluctuations can therefore alleviate trapping, but they do not reproduce the much larger enhancement generated by the embedding network.

### 3.4 Analytical theory of a noisy Boltzmann neuron

The mechanism underlying Recurrence Resonance can be examined analytically in a simplified system. We therefore consider a single Boltzmann neuron with self-coupling *w* and an optional bias *b* (Figure 2a). For analytical convenience, the Gaussian noise used in the network simulations is replaced by noise drawn uniformly from the interval [−*a*, +*a*]. Averaging over this noise broadens the effective neuronal response function (Figure 2b) and permits the information flux *I* to be calculated explicitly as a function of the coupling strength *w*, bias *b*, and noise half-width *a* (Section 2.7). This single-neuron model is not intended as a quantitative reduction of the ECM; rather, it isolates how coupling, noise, and bias jointly determine information flux.

**Figure 2:**
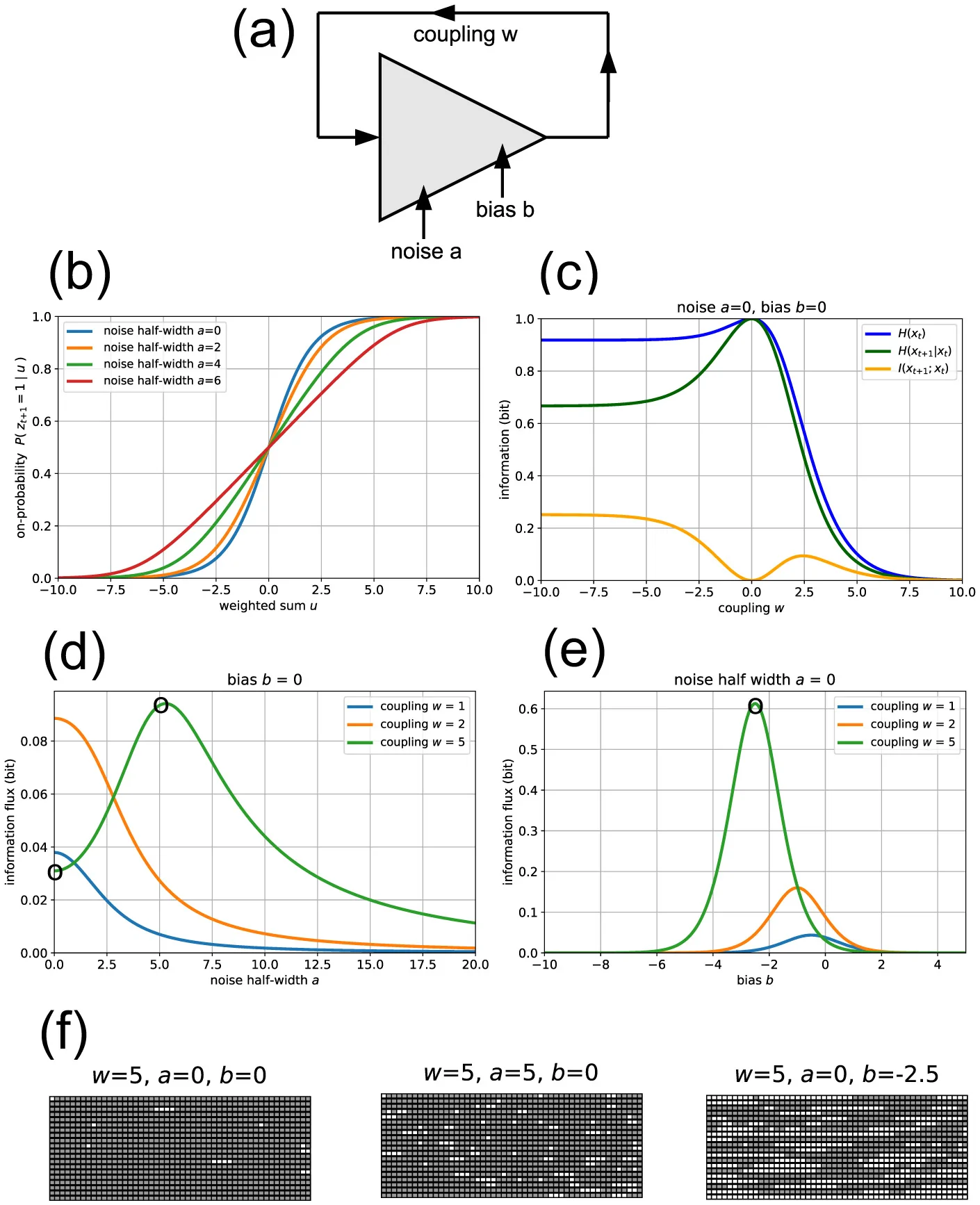
Analytical theory of a single noisy Boltzmann neuron. **(a)** Circuit diagram. **(b)** Effective response function for different noise half-widths *a*. **(c)** Entropy *H*(*x*_*t*_), conditional entropy *H*(*x*_*t*+1_|*x*_*t*_), and mutual information *I*(*x*_*t*_; *x*_*t*+1_) as functions of the recurrent self-coupling strength *w* in the absence of noise and bias. **(d)** Mutual information as a function of noise half-width *a* for three self-coupling strengths *w* without bias. **(e)** Mutual information as a function of bias *b* for three self-coupling strengths *w* without added noise. **(f)** Simulated time series of 1,000 successive binary activations of a strongly coupled neuron (*w* = 5): without noise or bias, with optimized noise, and with optimized bias. The corresponding operating points are marked by black circles in **(d, e)**. The parameters *w*, *b*, and *a* are expressed in units of the effective temperature.

We first consider the neuron without noise and bias while varying the self-coupling strength *w* (Figure 2c). The mutual information between successive activations can be written as *I*(*x*_*t*_; *x*_*t*+1_) = *H*(*x*_*t*+1_)−*H*(*x*_*t*+1_|*x*_*t*_). The entropy *H*(*x*_*t*+1_) quantifies the overall variability of the states visited by the system, whereas the conditional entropy *H*(*x*_*t*+1_|*x*_*t*_) quantifies the uncertainty that remains about the next state once the current state is known. High information flux consequently requires both diverse state occupation and sufficiently reproducible transitions.

For strongly negative coupling, the neuron tends to oscillate. A perfectly regular alternation between the on- and off-states cannot be achieved without bias, however, because after every off-state the next activation remains on with probability 1/2. This built-in indeterminism produces a relatively large conditional entropy (Figure 2c, green curve). Although the state entropy is close to one bit, the resulting information flux therefore remains limited to approximately *I* = 0.25 bit.

At *w* = 0, the unbiased Boltzmann neuron behaves as a binary random-number generator, with statistically independent states at successive time steps. Its entropy and conditional entropy are both one bit, and the information flux is zero. As *w* becomes positive, increasingly long sequences of on-states make the dynamics more predictable and reduce the conditional entropy. At the same time, the state distribution becomes progressively less balanced, causing the entropy to decrease as well. The difference between these two quantities produces a peak in information flux around *w* ≈ 2.5. For very large positive coupling, the neuron becomes trapped predominantly in the on-state and the information flux again approaches zero because it is bounded by the vanishing state entropy.

The analytical calculation reproduces the central qualitative feature of the network noise sweep: a resonance-like maximum as a function of noise amplitude occurs for strong positive coupling, where the noise counteracts an otherwise highly restricted state distribution (Figure 2d, green curve). For weaker coupling, where the unperturbed dynamics are not comparably trapped, increasing noise instead reduces information flux monotonically (Figure 2d, blue and orange curves). Recurrence Resonance is therefore not a universal benefit of noise, but depends on the operating regime of the recurrent system.

The same theory shows that a constant bias can provide a more effective alternative to added noise (Figure 2e). By shifting the response function rather than broadening it, an appropriate bias can balance state occupation without introducing an equally large stochastic contribution to the transitions. This effect is present even for coupling strengths at which added noise produces no resonance.

Simulated activation sequences of a neuron with strong excitatory self-coupling illustrate these mechanisms (Figure 2f). Without noise or bias, long on-periods are interrupted only occasionally by short off-periods, yielding a small information flux of approximately *I* = 0.03 bit. Optimized noise increases the frequency and duration of the off-periods and raises the information flux to approximately 0.095 bit. An optimized bias produces a substantially more balanced yet still temporally dependent sequence and increases the information flux to approximately 0.6 bit.

For a binary neuron, the theoretical maximum is one bit. It can be approached when sufficiently strong recurrent coupling makes transitions predictable while a suitable bias maintains balanced occupation of the two states. The analytical result thus anticipates a central question addressed below: whether the ECM enhances information flux primarily through fluctuating input or by shifting the operating points of the individual core neurons.

### 3.5 Simulated lesion experiments

To identify which directed connections are responsible for the flux-enhancing effect of the embedding network, we removed selected projection blocks between the core, periphery, and interneuron populations and compared each lesioned network with its intact counterpart (Figure 3). Because the comparisons are paired within network realizations, changes near zero indicate that the removed pathway is not required for maintaining the reference information flux.

**Figure 3:**
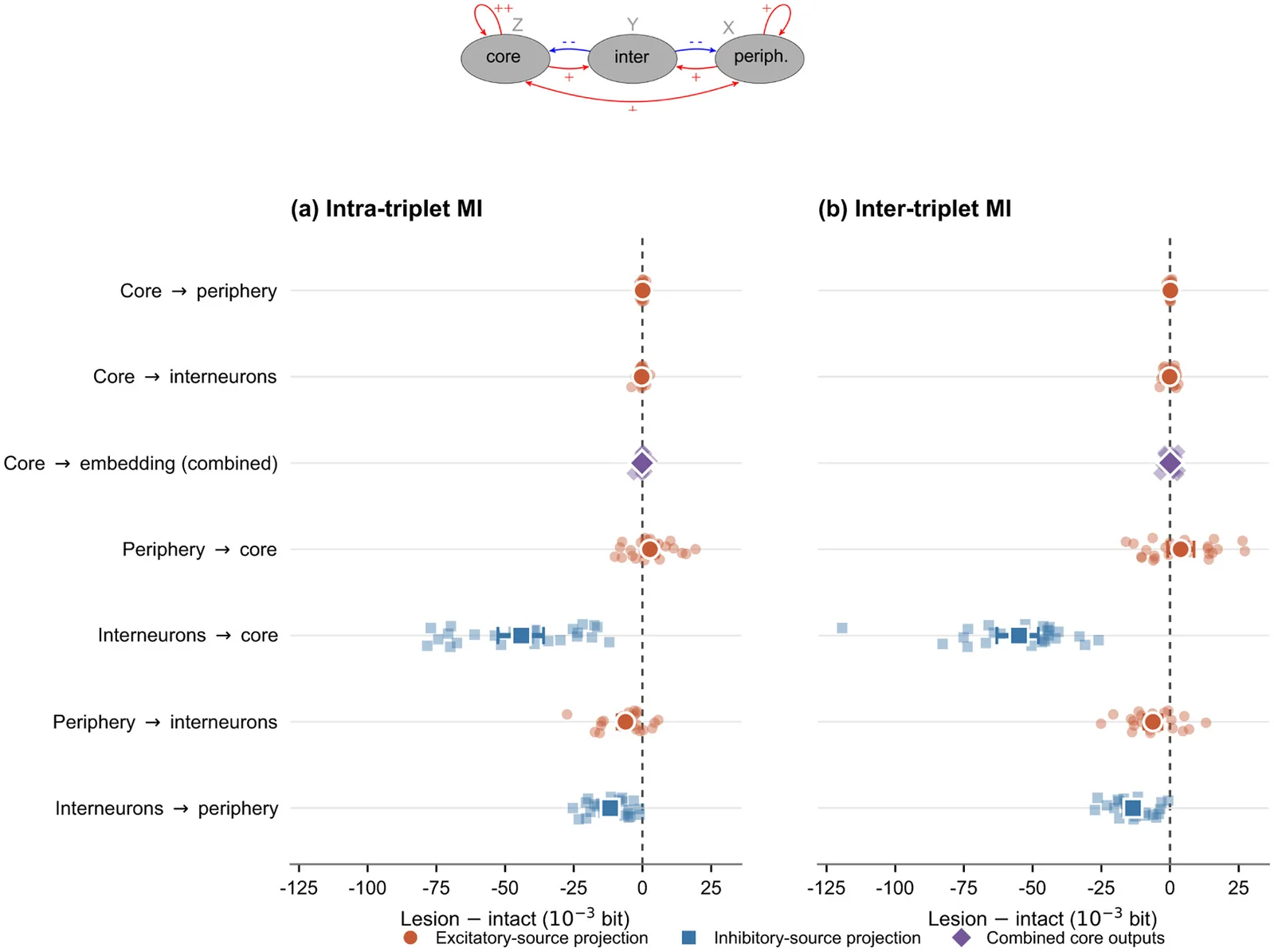
Ensemble lesion analysis of directional population-to-population projections. The unnumbered schematic reproduces the original population diagram and is included for orientation only. **(a, b)** Paired changes in intra-triplet and inter-triplet mutual information, respectively, after removing the indicated directed projection block, relative to the intact version of the same network. Small symbols show the 25 individual networks; large symbols and whiskers show the ensemble mean and its percentile-bootstrap 95% confidence interval (20,000 resamples across networks). Negative values indicate reduced mutual information after the lesion. Colors and symbol shapes distinguish lesions of excitatory-source projections, inhibitory-source projections, and the combined core-output lesion. Core to embedding (combined) denotes simultaneous removal of the core-to-periphery and core-to-interneuron projections.

Removing projections from the core to the surrounding populations had essentially no ensemble-level effect. For the core-to-periphery lesion, the mean changes were +0.10 × 10^−3^ bit for intra-triplet mutual information and +0.15 × 10^−3^ bit for inter-triplet mutual information; for the core-to-interneuron lesion, they were −0.28 × 10^−3^ bit and −0.11 × 10^−3^ bit, respectively. All four confidence intervals included zero (Figures 3a, b). Simultaneously removing both sets of core outputs likewise produced changes near zero. Thus, the embedding network does not require moment-to-moment input from the core to sustain its flux-enhancing action, arguing against a closed regulatory feedback loop as the principal mechanism.

The converse lesions revealed a strongly asymmetric dependence. Removing inhibitory projections from the interneurons to the core reduced intra-triplet mutual information by a mean of 44.1 × 10^−3^ bit (95% confidence interval: 36.0–52.6 × 10^−3^ bit) and inter-triplet mutual information by 55.0 × 10^−3^ bit (47.9–63.0 × 10^−3^ bit). Both effects were negative in all 25 network realizations and were much larger than those of any other lesion. Direct inhibitory input from the interneuron population is therefore the dominant pathway preventing the core from entering its highly active, low-flux regime.

By contrast, removing the direct excitatory projection from the periphery to the core did not consistently reduce information flux. The ensemble-mean changes were slightly positive, +2.72 × 10^−3^ bit for the intra-triplet measure and +3.88 × 10^−3^ bit for the inter-triplet measure, but varied substantially across networks and both confidence intervals included zero. The ensemble therefore provides no evidence that this direct pathway is itself required for flux enhancement.

Interactions within the embedding network nevertheless contributed reproducibly. Removing projections from the periphery to the interneurons decreased intra-triplet mutual information by 6.16 × 10^−3^ bit (95% confidence interval: 3.36–9.20 × 10^−3^ bit) and inter-triplet mutual information by 6.22 × 10^−3^ bit (2.87–9.55 × 10^−3^ bit). Removing projections from the interneurons to the periphery produced larger decreases of 11.76 × 10^−3^ bit (9.00–14.50 × 10^−3^ bit) and 13.46 × 10^−3^ bit (10.70–16.22 × 10^−3^ bit), respectively, and reduced both measures in every network realization. The periphery thus appears to support information flux chiefly as part of the surrounding circuit rather than through its direct excitation of the core.

Input-resolved measurements further clarified these two embedding-network lesions (Supplementary Figures S4a–c). Removing periphery-to-interneuron projections increased the mean embedding input to the core by 1.554 (95% confidence interval: 1.457–1.651) and increased mean core activity by 0.0912 (0.0831–0.1001); the accompanying fluctuations became larger rather than smaller. Its adverse information-flux effect is therefore primarily consistent with a shift of the mean operating point. Removing interneuron-to-periphery projections likewise increased the mean embedding input by 1.435 (1.225–1.629) and core activity by 0.0810, but additionally reduced input variance and covariance. This lesion therefore combines an unfavorable operating-point shift with a loss of shared fluctuations.

Taken together, these lesions separate the enhancement of information flux from feedback control by the core. The embedding populations can generate beneficial input without receiving ongoing core output, but their internal interaction helps establish the regime from which the interneurons deliver the crucial inhibitory signal. The remaining question is whether the benefit of this signal lies mainly in its mean level, acting as a bias, or in its temporal fluctuations. We next characterize the activity and statistical dependencies within the intact ECM before addressing that distinction directly.

### 3.6 Neural activations and pairwise mutual information

We next examined activity and pairwise statistical dependencies within the intact ECM. A representative raster plot illustrates the heterogeneous activation patterns of the three populations (Figure 4a). Across the 25 networks, the mean on-probability was 0.548 in the core (95% confidence interval: 0.509–0.585), 0.273 in the periphery (0.270–0.276), and 0.612 in the interneuron population (0.609–0.615). Thus, the reference regime combines an approximately balanced core with a less active periphery and a more active inhibitory population.

**Figure 4:**
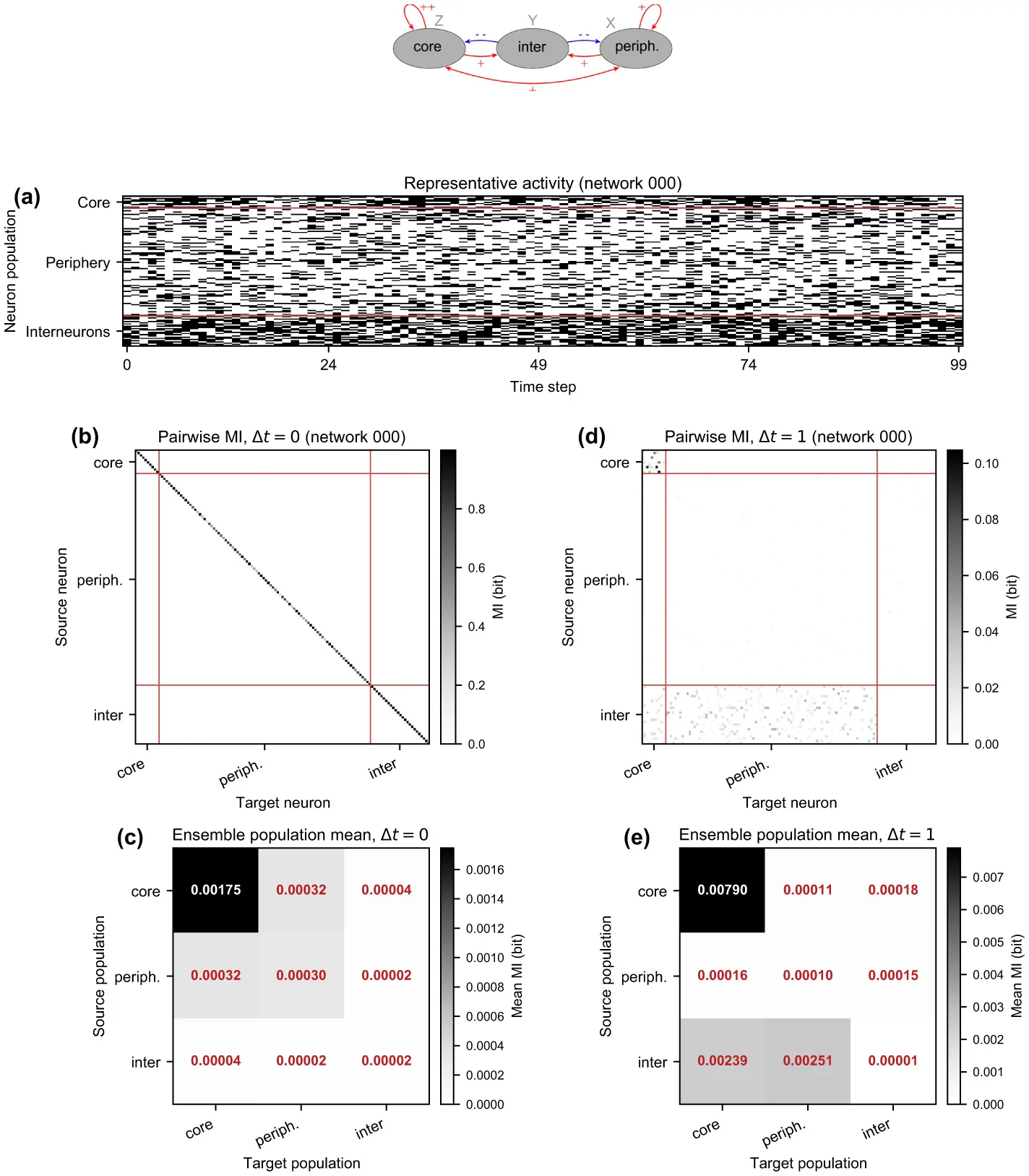
Activation statistics and pairwise mutual information in the embedded network. The unnumbered schematic is included for population-level orientation. **(a)** Binary activity of all 125 neurons over 100 successive time steps for representative network 000 and dynamics seed 10,001. **(b, d)** Complete 125 × 125 matrices of simultaneous pairwise mutual information (Δ*t* = 0) and one-step time-lagged pairwise mutual information (Δ*t* = 1), respectively, for the same representative trajectory. Rows denote source neurons and columns denote target neurons. The full matrices retain their diagonal for orientation. **(c, e)** Corresponding 3 × 3 population-level matrices, averaged first across the two dynamics seeds within each network and then across all 25 independently generated networks. When source and target belong to the same population, only distinct-neuron pairs (*i* ≠ *j*) enter these averages; self-pairs are excluded. Numerical entries give ensemble-mean mutual information in bits. The time-lagged measure quantifies temporal statistical dependence and is not interpreted as a causal information-transfer measure.

Shared inputs and recurrent interactions can produce statistical dependencies between pairs of simultaneously observed neurons. The complete matrix *I*(*x*_*t*_; *y*_*t*_) for a representative network contains large diagonal entries because these entries are the entropies of individual neurons (Figure 4b). To characterize dependencies between distinct neurons, we excluded these self-pairs from the population-level averages shown in Figure 4c. The largest simultaneous pairwise mutual information then occurred within the core, with an ensemble mean of 1.75 × 10^−3^ bit (95% confidence interval: 1.48–2.07 × 10^−3^ bit). The next-largest means were found between core and periphery (0.321 × 10^−3^ bit) and within the periphery (0.300 × 10^−3^ bit), whereas all pairings involving interneurons were substantially smaller. The strong within-core dependence is consistent with the dense recurrent coupling of that population.

The one-step time-lagged mutual information *I*(*x*_*t*_; *y*_*t*+1_) showed a more strongly directional pattern (Figures 4d, e). The largest population-level mean was again found recursively within the core, at 7.90 × 10^−3^ bit. This was followed by dependencies from the interneurons to the periphery (2.51 × 10^−3^ bit) and from the interneurons to the core (2.39 × 10^−3^ bit). In the reverse direction, the means from the core to the periphery and to the interneurons were only 0.108 × 10^−3^ bit and 0.178 × 10^−3^ bit, respectively. This asymmetry is consistent with the lesion results: interneuron outputs are closely associated with subsequent activity in their target populations, whereas core outputs are not required for the embedding network to maintain high core information flux.

Pairwise mutual information measures statistical dependence and can reflect direct connections, indirect pathways, common input, and population-wide state changes. In particular, the time-lagged measure should not be interpreted as a causal information-transfer estimate. Nevertheless, its population pattern complements the intervention-based lesion analysis and motivates a more direct examination of the signals that the embedding network delivers to individual core neurons.

### 3.7 Signals from embedding networks to the core

We next characterized the signals that the core neurons receive from the periphery and interneuron populations. For each core neuron *i*, we recorded the embedding contribution *u*_*i*,emb_(*t*) to its total synaptic input. The resulting neuron-specific distributions are shown for representative network 000 in Figure 5a. Because each core neuron receives projections from only a subset of binary embedding neurons with fixed synaptic weights, its input can assume a finite set of underlying values. Kernel-density visualization produces continuous curves, but their pronounced multimodal structure reflects this discrete origin.

**Figure 5:**
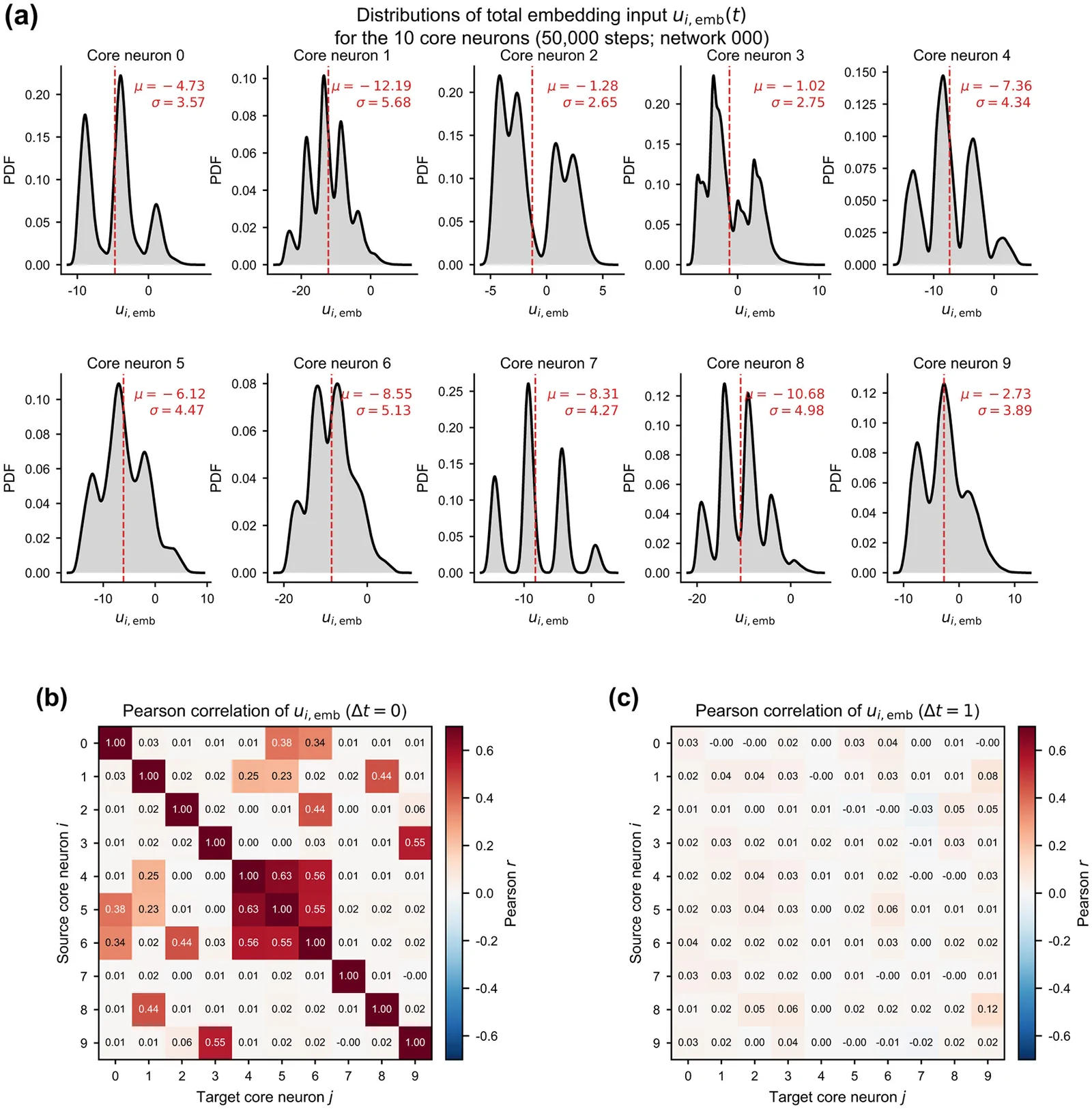
Analysis of signals from the embedding network to the core. **(a)** Probability-density estimates of the total synaptic input *u*_*i*,emb_(*t*) from the periphery and interneuron populations, shown separately for the ten core neurons of representative network 000. The estimates use 50,000 post-burn-in samples and Gaussian kernels with a bandwidth equal to 0.1 times the sample standard deviation. Red dashed lines mark the neuron-specific temporal means; the corresponding mean μ and standard deviation σ are reported in each panel. **(b)** Pairwise instantaneous Pearson correlations between the embedding inputs received by the core neurons. **(c)** Corresponding one-step time-lagged correlations, with rows denoting source signal *u*_*i*,emb_(*t*) and columns denoting target signal *u*_*j*,emb_(*t* + 1). **(b, c)** use the same representative trajectory and color scale. The panels provide a detailed example; across-network summaries of mean and maximum absolute correlations for all 25 network realizations accompany the plotting code.

The temporal mean μ_*i*_ and standard deviation σ_*i*_ of each distribution provide first-order descriptions of a constant neuron-specific bias and the amplitude of the accompanying fluctuations. In the representative network, all ten means were negative and ranged from −12.19 to −1.02, while the standard deviations ranged from 2.65 to 5.68 (Figure 5a). The same tendency was robust but not universal across the ensemble: 228 of the 250 neuron–network combinations had negative means, and |μ_*i*_| > σ_*i*_ in 193 cases. Averaged over all core neurons and networks, μ_*i*_ was −6.37 and σ_*i*_ was 4.01. Thus, the embedding input generally contains a substantial negative mean component, but its magnitude and even its sign vary across individual neurons and network realizations.

The input fluctuations were also not independent across core neurons. In the representative example, several instantaneous off-diagonal Pearson correlations were pronounced and predominantly positive (Figure 5b). Across the 25 networks, the mean absolute instantaneous off-diagonal correlation was 0.107 (95% confidence interval: 0.096–0.117), and the mean of the largest absolute correlation within each network was 0.583 (0.536–0.633). Such spatial correlations can arise when different core neurons receive input from overlapping or statistically coupled subsets of the embedding network.

Projection-overlap analysis supported this structural interpretation (Supplementary Figures S4d–f). Across pairs of core targets, the Jaccard overlap of presynaptic source sets predicted their embedding-input covariance both for periphery projections (*r* = 0.522, 95% confidence interval: 0.461–0.578) and, especially, for inhibitory projections (*r* = 0.857, 0.794–0.924). The association was positive within individual networks as well. Shared fluctuations are therefore not merely a fitted property of the input traces but arise systematically from overlapping embedding projections.

One-step time-lagged correlations were considerably weaker (Figure 5c). Their ensemble-mean absolute value was 0.0227 (95% confidence interval: 0.0213–0.0240), although the largest absolute lag-one correlation within a network averaged 0.134 (0.116–0.152). The embedding signals can therefore be regarded as only weakly correlated across successive time steps, but not as perfectly white or spatially independent noise.

These observations identify several signal properties that may contribute to information flux. They include neuron-specific means and fluctuation amplitudes. The signals also exhibit covariance across neurons, non-Gaussian marginal structure, and residual temporal dependence. Descriptive statistics alone cannot establish which of these properties is functionally important. We therefore separate the components in the replacement experiments below.

### 3.8 Relevance of biases and fluctuations

We next asked whether the flux-enhancing effect of the embedding signals is attributable mainly to their neuron-specific temporal means or to their fluctuations. To separate these contributions, we removed the surrounding populations while retaining the same recurrent core and replaced the embedding input by signals that preserved selected empirical properties (Figure 6). The noise-free isolated core again provided the low-flux reference, with a mean indicator of *f* = 30.7, compared with *f* = 116.0 under full embedding.

**Figure 6:**
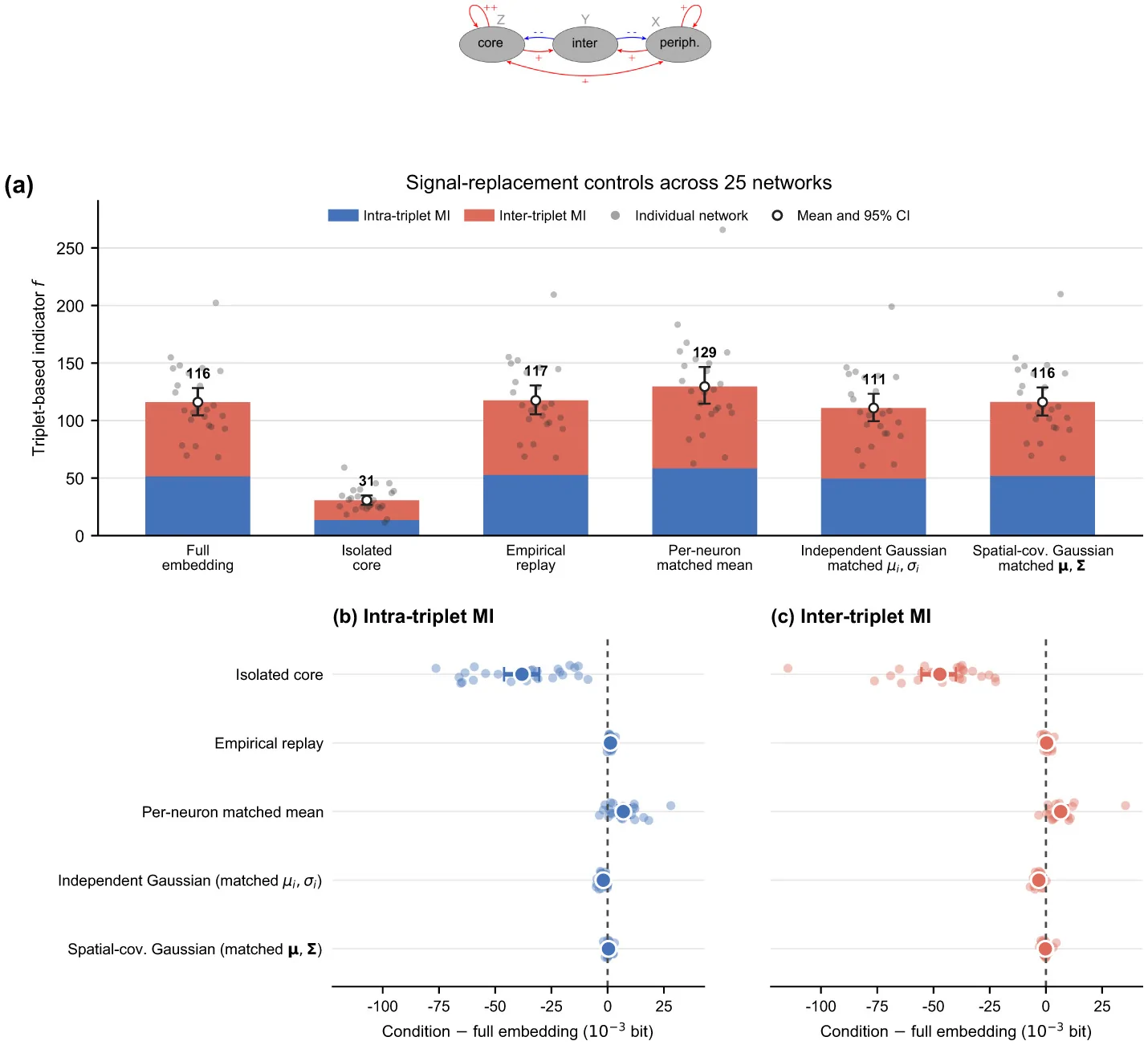
Ensemble comparison of embedding-signal replacement controls. The unnumbered schematic is included for population-level orientation. **(a)** Absolute triplet-based information-flux indicator under full embedding, isolation, empirical input replay, replacement by the per-neuron matched mean, independent Gaussian input matched to each neuron's empirical mean and standard deviation, and spatial-covariance-matched Gaussian input matched to the empirical mean vector and instantaneous covariance matrix. Stacked bars show the ensemble means of the intra-triplet and inter-triplet contributions; small points show total indicator values for the 25 individual networks, and open symbols with whiskers show ensemble means and bootstrap 95% confidence intervals. Numbers above the bars give mean total indicator values. **(b, c)** Within-network changes in intra-triplet and inter-triplet mutual information, respectively, relative to full embedding. Small points show individual paired changes; large symbols and whiskers show their ensemble means and percentile-bootstrap 95% confidence intervals based on 20,000 resamples of network identifiers. Negative values indicate lower mutual information than under full embedding. Empirical replay preserves the recorded vector input sequence but removes ongoing feedback. Neither Gaussian control was designed to reproduce temporal covariance.

As a first control, we replayed the complete recorded vector sequence **u**_emb_(*t*) to the isolated core. Replay preserves the realized means, marginal distributions, spatial correlations, and temporal ordering of the embedding input, but removes ongoing interaction with the surrounding populations. It closely reproduced the full embedded condition, yielding a mean indicator of *f* = 117.5 (Figure 6a). Relative to full embedding, the mean paired changes were only +1.24 × 10^−3^ bit for the intra-triplet contribution and +0.30 × 10^−3^ bit for the inter-triplet contribution (Figures 6b, c). This agreement reinforces the lesion result that moment-to-moment feedback from the core is not required once the embedding signal has been generated.

We then replaced the entire time-dependent signal received by each neuron with its own empirical temporal mean μ_*i*_. These per-neuron matched means were measured from the corresponding embedded network and were not optimized. Nevertheless, they produced a mean indicator of *f* = 129.5, exceeding full embedding in 23 of the 25 paired networks. The mean increases were 6.96 × 10^−3^ bit for intra-triplet mutual information (95% confidence interval: 4.35–9.85 × 10^−3^ bit) and 6.57 × 10^−3^ bit for inter-triplet mutual information (4.23–9.63 × 10^−3^ bit). Thus, the neuron-specific mean inputs alone explain the enhancement relative to isolation and, on average, place the core in a still higher-flux regime than the complete fluctuating signals.

Restoring fluctuations as temporally white, mutually independent Gaussian signals matched to each neuron's μ_*i*_ and σ_*i*_ reduced the mean indicator to *f* = 110.9. This value remained far above the isolated-core result but was lower than full embedding in every network realization. The paired decreases were 1.92 × 10^−3^ bit for the intra-triplet contribution (95% confidence interval: 1.33–2.55 × 10^−3^ bit) and 3.15 × 10^−3^ bit for the inter-triplet contribution (2.51–3.80 × 10^−3^ bit). Independent Gaussian fluctuations therefore do not add to the benefit of the already favorable means; instead, they cause a modest loss.

This loss does not by itself demonstrate an advantage of the non-Gaussian embedding-input distributions, because the independent Gaussian control also removes the spatial correlations identified in Section 3.7. We therefore generated multivariate Gaussian signals matched to the complete empirical mean vector **μ** and instantaneous covariance matrix **Σ**. This spatial-covariance-matched control restored the mean indicator to *f* = 116.1, essentially identical to the embedded value. Its paired differences from full embedding were +0.37 × 10^−3^ bit for the intra-triplet contribution and −0.25 × 10^−3^ bit for the inter-triplet contribution, and both confidence intervals included zero.

Taken together, the replacement experiments identify the neuron-specific means as the principal flux-enhancing component of the embedding signals. Fluctuations are not required to explain the improvement over isolation, and independent fluctuations are slightly detrimental once these means are present. Accounting for instantaneous spatial covariance is sufficient for Gaussian signals to reproduce the full embedded result. We therefore find no evidence that non-Gaussian marginal structure provides a specific advantage under the tested conditions. The next question is whether the empirically generated means are themselves optimal or whether simpler bias regulation can move the core into an even higher-flux regime.

### 3.9 Optimization of biases

The per-neuron matched means were generated by the embedding network rather than selected to maximize information flux. We therefore returned to the isolated core and first swept a single uniform bias *b*_*i*_ = *b* across all ten neurons (Figure 7b). The ensemble-mean training indicator followed a broad non-monotonic curve with its largest sampled value, *f* = 187.5, at *b* = −7.5. Network-specific maxima occurred at slightly different values, with a mean selected bias of −7.34. When these individually selected uniform biases were evaluated on held-out trajectories, the ensemble-mean indicator was *f* = 191.6 (95% confidence interval: 173.3–211.3), compared with *f* = 116.1 under full embedding (Figure 7a). The uniform-bias condition outperformed the embedded condition in every one of the 25 paired networks.

**Figure 7:**
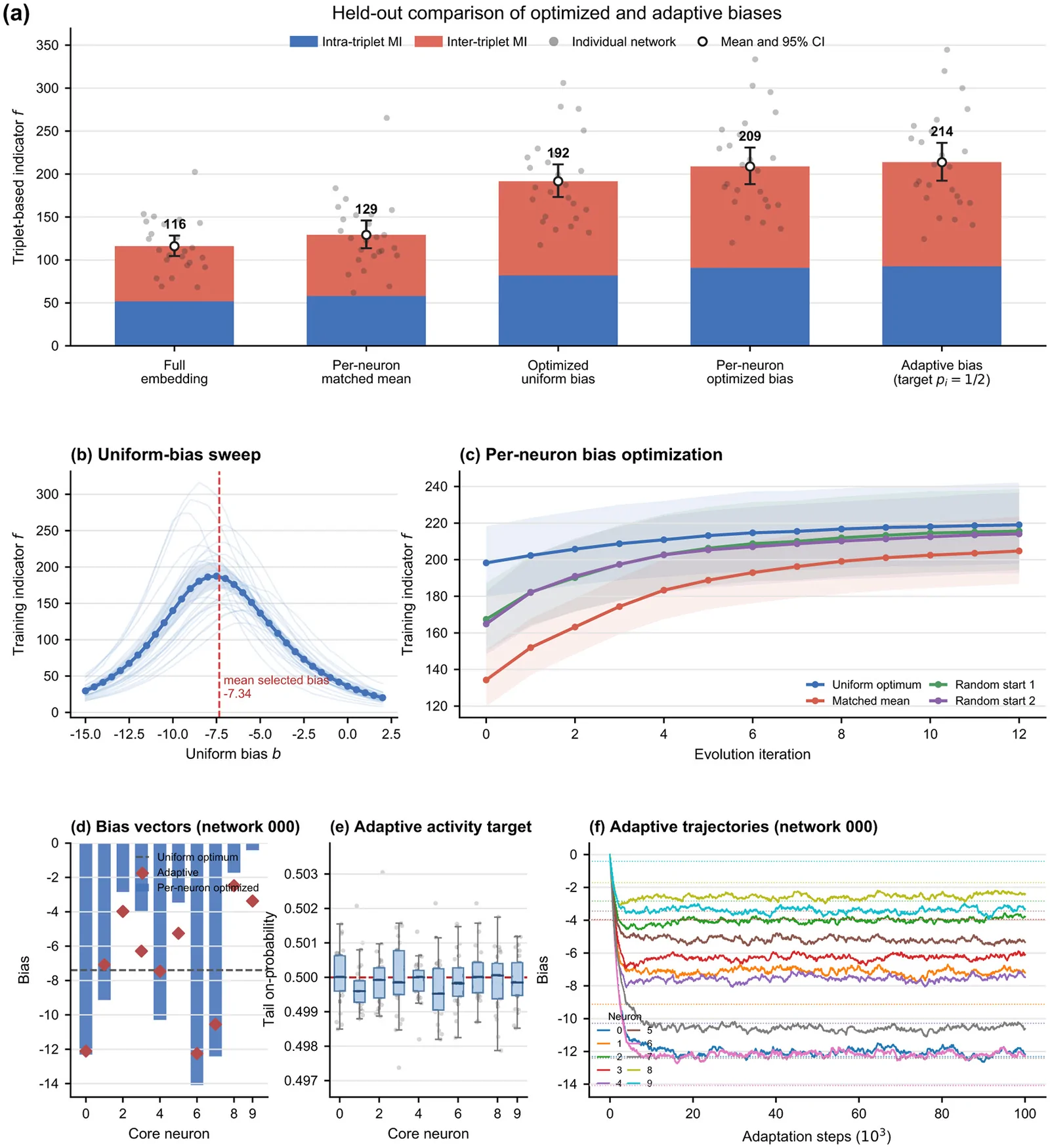
Numerical optimization and local adaptation of core biases. **(a)** Held-out comparison of the complete embedded network, the isolated core driven by its per-neuron matched empirical means, the optimized uniform bias, the per-neuron optimized bias vector, and the locally learned adaptive bias. Stacked bars show ensemble-mean intra-triplet and inter-triplet contributions to the indicator; small points show the 25 individual networks, and open symbols with whiskers show ensemble means and bootstrap 95% confidence intervals. Bias conditions were selected independently of the two held-out evaluation seeds. Adaptive values were averaged over two adaptation seeds and two held-out evaluation seeds within each network. **(b)** Coarse uniform-bias search. Pale curves show individual networks; the dark curve and band show the ensemble mean and bootstrap 95% confidence interval. The dashed line marks the mean of the 25 network-specific selected uniform biases. **(c)** Ensemble-mean training objective during the fixed-budget per-neuron evolutionary search for its four starting conditions; shaded regions show bootstrap 95% confidence intervals. **(d)** Numerically optimized and adaptively learned neuron-specific biases for representative network 000; the dashed horizontal line marks its optimized uniform bias. **(e)** Distribution across the 25 networks of the final mean activity of each core neuron after local bias adaptation. Values were first averaged across the two adaptation seeds within each network; the dashed line marks the target on-probability one half. **(f)** Bias trajectories for representative network 000 and adaptation seed 51,001. Dotted horizontal lines of matching color show that network's numerically optimized per-neuron biases. **(b, c)** show training objectives, whereas **(a)** reports independent held-out evaluations.

Allowing each core neuron to receive its own constant bias provided a further improvement. Starting from four different bias vectors, the fixed-budget evolutionary searches increased their training objectives and approached similar high-flux regimes (Figure 7c). The selected per-neuron bias vectors produced a held-out ensemble mean of *f* = 208.8 (95% confidence interval: 188.2–230.8). They outperformed full embedding in all 25 networks and the corresponding optimized uniform bias in all 25 networks, with a mean additional increase of 17.2 indicator units.

The selected biases were heterogeneous across neurons, as illustrated for network 000 in Figure 7d, where they ranged from −14.08 to −0.41. This heterogeneity is consistent with the different recurrent inputs received by the individual core neurons. Because the evolutionary procedure used a finite search budget, these vectors represent the best solutions found by the specified search rather than guaranteed global optima. The held-out improvement nevertheless establishes that neuron-specific static biases can place every tested core in a substantially higher-flux regime than its complete embedding network.

We finally asked whether a local adaptive mechanism could reach a similarly favorable operating point without directly evaluating information flux. Each neuron independently adjusted its bias to drive its time-averaged on-probability toward 1/2. From zero initialization, the biases rapidly became negative and stabilized at heterogeneous neuron-specific levels (Figure 7f). Across the ensemble, the final mean activities of all ten neuron identities were extremely close to the target, with ensemble means between 0.4997 and 0.5001 and all individual network means between 0.4974 and 0.5031 (Figure 7e).

After adaptation was stopped and the learned biases were held fixed, the mean indicator on independent evaluation trajectories was *f* = 213.8 (95% confidence interval: 192.3–236.3). The adaptive condition exceeded full embedding in all 25 networks and was slightly higher than the numerically optimized per-neuron condition in 24 of 25 networks, by 5.0 indicator units on average (Figure 7a). The adaptive and numerically selected bias vectors were similar in scale but not identical (Figures 7d, f). Thus, convergence to an on-probability of one half does not imply convergence to a unique bias vector.

The target *p*_*i*_ = 1/2 maximizes the marginal output entropy of each binary neuron, but it does not mathematically guarantee maximal mutual information, which also depends on conditional entropy and joint network dynamics. Its strong performance nevertheless shows that a simple local activity-homeostasis rule can discover a high-flux regime without access to the global information measure. More broadly, these results distinguish enhancement from optimization: the embedding network robustly rescues information flux relative to isolation, but neither its architecture nor its empirically generated biases maximize the indicator within the tested alternatives.

Decomposing the signal-replacement and bias results into successor-state entropy and conditional entropy showed that both terms generally changed together (Supplementary Figure S1 and Supplementary Table S1). Isolation reduced the successor-state entropy by 1.164 bit; a nearly as large reduction in conditional entropy partly compensated for this loss but did not prevent the decrease in mutual information. The per-neuron matched means reduced both quantities more modestly, with the larger reduction in conditional entropy producing a net flux increase. Conversely, optimized and adaptive biases increased both state entropy and transition uncertainty, but their entropy gains were larger and therefore yielded higher mutual information. Independent Gaussian input produced a small unfavorable balance of the two terms, whereas the component changes under spatial-covariance-matched Gaussian input nearly canceled. Thus, neither biases nor noise act exclusively through one information-theoretic component; their effect is determined by the balance Δ*I* = Δ*H* − Δ*H*_cond_.

### 3.10 Robustness and sensitivity of the embedded operating regime

The preceding bias analyses were performed in isolated cores. We therefore also applied the local adaptive rule while the complete embedding network remained present. Adaptation increased the mean indicator from 116.07 to 177.04, a paired gain of 60.97 (95% confidence interval: 46.44–76.30) observed in all 25 networks, while bringing the mean tail activity to 0.49993 (Supplementary Figure S2). The adaptive embedded condition nevertheless remained below the adaptive isolated-core condition by 36.76 indicator units (31.28–42.82, expressed as the advantage of isolated adaptation). Thus, embedding does not prevent local homeostatic regulation, but the fixed embedding input constrains the operating point that regulation can reach.

We next relaxed the assumption that all inhibitory projections share one common weight. Across the tested two-dimensional grid, the common ensemble optimum combined an interneuron-to-core weight of −5 with a stronger interneuron-to-periphery weight of −30. Relative to the common-weight reference, this increased the mean indicator by 6.90 (95% confidence interval: 4.84–8.98) and improved 22 of 25 networks. Selecting the best tested pair separately for each network increased the mean gain to 13.87 (10.68–17.12) and improved all 25 networks (Supplementary Figure S3). The equal-weight baseline is therefore a useful conventional reference, but not the optimum of the broader inhibitory parameterization.

The benefit of embedding also depended decisively on the assignment of strong recurrent core weights (Supplementary Figures S5c–f). With the ranked assignment used throughout the main analyses, embedding increased the mean indicator by 85.08 (95% confidence interval: 73.16–98.81), with positive effects in all 25 networks. After globally randomizing the core-weight assignment, the mean effect was instead −3.80 (−4.34 to −3.27), and after targeted swaps that removed the preferential concentration of large weights from the designated core skeleton it was −2.81 (−3.28 to −2.37); neither control produced a positive effect in any network. These manipulations are architectural sensitivity controls rather than biological null models. They show that strong core recurrence is the dynamical substrate whose low-flux operating point is rescued by embedding.

Finally, we repeated the principal uncertainty calculations using percentile and bias-corrected-and-accelerated bootstrap intervals, paired *t* intervals, sign-flip tests, and leave-one-network-out estimates (Supplementary Figure S6). The direction of 16 of 20 prespecified contrasts was stable across all procedures, and the remaining differences concerned contrasts already close to zero rather than the central embedding, inhibition, or bias effects. These checks support the qualitative conclusions while retaining the independently generated network as the statistical unit. Having established both the robustness and the architectural limits of the operating-regime effects, we next examine whether the reduced indicator tracks full-state mutual information.

### 3.11 Validation of the triplet-based information-flux indicator

The preceding ensemble analyses used the triplet-based indicator as a computationally tractable summary of temporal dependence. We therefore tested directly whether this reduced measure tracks the mutual information between successive states of the complete 10-neuron core. For five independently generated cores, we compared the triplet indicator with semianalytical full-state mutual information across 36 uniform-bias conditions per network. Both measures displayed closely corresponding non-monotonic profiles, increasing from low values at strongly negative biases, reaching a maximum at an intermediate bias, and declining again at less negative and positive biases (Figures 8a, b).

**Figure 8:**
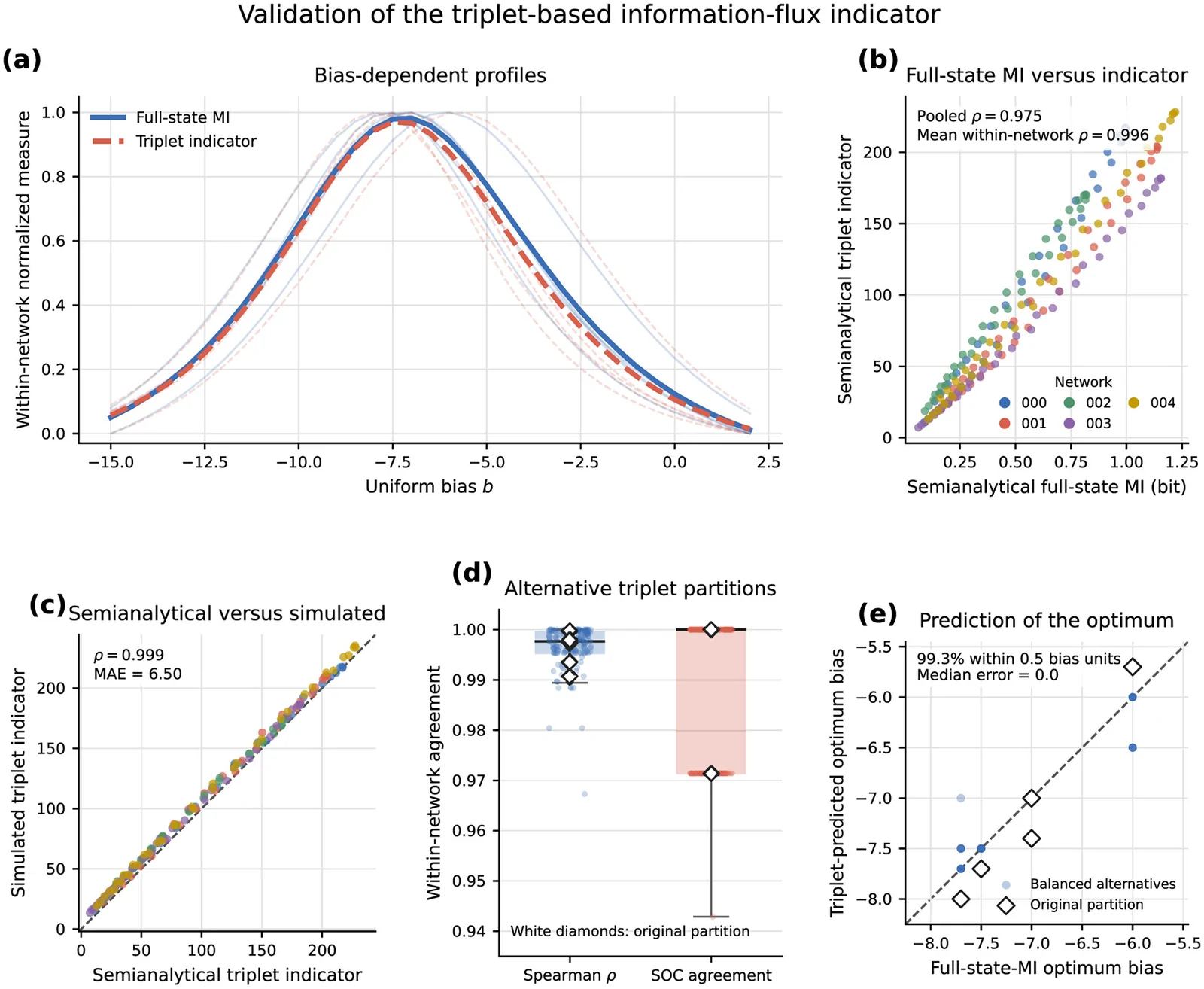
Validation of the triplet-based information-flux indicator against the semianalytical mutual information of the complete 10-neuron core. **(a)** Bias-dependent profiles of full-state mutual information and the triplet indicator for five independently generated networks. Pale lines show individual networks; dark lines show means across networks. Both quantities were normalized separately within each network to compare their shapes without imposing a common scale. **(b)** Semianalytical full-state mutual information vs. the semianalytical triplet indicator for all 180 evaluated network-bias points. Colors identify networks. The pooled and mean within-network Spearman correlations are reported in the panel. **(c)** Semianalytical vs. simulation-based estimates of the triplet indicator at the same points. The dashed line denotes equality; MAE is the mean absolute error in indicator units. **(d)** Robustness to triplet definition. Points summarize 150 balanced alternative partitions, comprising 30 partitions for each network and omitting each of the ten core neurons in turn; boxes show their distributions. White diamonds denote the original partition used in the main analyses. SOC agreement is the fraction of consecutive bias intervals for which full-state mutual information and the triplet indicator change in the same direction. **(e)** Bias maximizing the triplet indicator vs. the bias maximizing full-state mutual information for the alternative and original partitions. The dashed line denotes equality. Together, the panels show that the triplet indicator reproduces the bias-dependent ordering and locates the full-state-MI optimum robustly despite using only low-dimensional marginal distributions.

Within individual networks, Spearman correlations between full-state mutual information and the triplet indicator ranged from 0.9907 to 0.9997, with a mean of 0.9959. The two measures changed in the same direction over a mean fraction of 0.9771 of successive bias intervals. Most importantly for the optimization experiments, the bias maximizing the triplet indicator closely approximated that maximizing full-state mutual information: the absolute difference ranged from 0 to 0.4 bias units and averaged 0.24 across the five networks. Thus, the reduced indicator preserved both the ordering of operating points and the location of the full-state optimum, despite not sharing the absolute scale of the full-state quantity.

We also compared the semianalytical triplet values with the corresponding estimates from finite simulated trajectories (Figure 8c). Across all 180 network–bias points, their Spearman correlation was 0.9992. The simulation estimates were slightly higher, with a mean absolute difference of 6.50 indicator units, but reproduced the ordering and profile of the semianalytical values. This agreement shows that the trajectory lengths used in the simulations were sufficient for the comparative analyses performed here.

Finally, we tested whether the validation depended on the original choice of neurons 0–8 and the omission of neuron 9. Thirty alternative partitions were examined for each network, with balanced omission of each of the ten core neurons. Across these 150 network-specific alternative partitions, the median Spearman correlation with full-state mutual information was 0.9977 and even the minimum was 0.9673 (Figure 8d). The median sign-of-change agreement was 1.000, with a minimum of 0.9429. The median error in the maximizing bias was zero, and 99.3% of the alternative partitions located the optimum within 0.5 bias units of the full-state result (Figure 8e). The original partition fell within the same high-agreement range.

These results validate the triplet-based indicator for the role assigned to it in this study: a low-dimensional measure for comparing dynamical regimes and identifying changes in information flux. They do not imply that its numerical value equals the full-state mutual information or that every possible manipulation must preserve the same relation. Within the tested bias-dependent operating regimes, however, the indicator reliably tracks the complete-core quantity, locates its optimum, and is robust to the particular triplet partition.

## 4 Discussion

### 4.1 Summary

In this work, we implemented and analyzed a simplified model of a cortical layer 5 microcircuit in which a small population of strongly connected excitatory neurons is embedded within larger excitatory and inhibitory populations. The central experiments were repeated across 25 independently generated network realizations. In every realization, the embedded core exhibited substantially greater information flux than the corresponding isolated core. Isolation left the strongly excitatory core in a highly active regime with restricted state occupation, whereas the surrounding populations reproducibly moved it into a more informative operating range.

The lesion experiments did not support the initially plausible hypothesis that this regulation requires a closed feedback loop through the embedding network. Removing projections from the core to the surrounding populations had essentially no effect on core information flux. In the opposite direction, inhibitory input from the interneuron population to the core was the dominant pathway, while interactions within the embedding populations also contributed. The embedding circuit can therefore generate beneficial input without requiring moment-to-moment feedback from the core.

The replacement experiments identified the neuron-specific temporal means of the embedding inputs as their principal flux-enhancing component. Applying these matched means as constant biases to the isolated cores was sufficient to explain the improvement over isolation and, on average, produced greater information flux than the complete embedding signals. Independent Gaussian fluctuations added to the same means caused a modest reduction. However, Gaussian signals additionally matched to the instantaneous spatial covariance reproduced the embedded result. We therefore found no evidence that non-Gaussian marginal statistics confer a specific advantage under the tested conditions.

The embedding-generated means were beneficial but not optimal among the tested alternatives. Network-specific uniform biases and neuron-specific bias vectors both outperformed the complete embedding network in all 25 realizations. A local adaptive rule that drove each neuron's mean on-probability toward one half reached a similarly high regime without evaluating the global information-flux objective. The entropy decomposition showed that these effects cannot be assigned exclusively to either state entropy or conditional entropy: both generally changed together, and the resulting mutual-information difference was determined by their balance.

The supplementary robustness analyses sharpened these conclusions. Adaptive regulation also improved the fully embedded core, and independently tuning inhibition to the core and periphery improved upon the common-weight baseline. At the same time, the embedding advantage disappeared when the preferential assignment of strong recurrent weights to the core was removed. Thus, the strong excitatory core is not incidental to the effect: it creates the operating-point problem that inhibitory embedding and bias regulation solve. The principal conclusions were insensitive to alternative interval estimates and paired statistical tests.

Finally, the triplet-based indicator used for the ensemble experiments closely tracked semianalytical full-state mutual information in the complete 10-neuron core. It preserved the bias-dependent ordering and location of the full-state optimum across the validation networks and remained robust under alternative triplet partitions. Together, these results establish a narrower but better-supported conclusion than the original optimization hypothesis: embedding robustly enhances information flux by regulating the core's operating point, but the architecture does not maximize information flux within the alternatives examined here.

### 4.2 Implications of the results

The finding that simple optimized biases outperform the complete embedding network does not make the surrounding circuit functionally irrelevant. It changes the interpretation of that function. The embedding architecture is better viewed as a distributed regulator that reproducibly prevents a strongly recurrent excitatory core from becoming trapped in a saturated, low-flux regime. A biological circuit may benefit more from maintaining a broad and robust operating range across heterogeneous neurons and changing conditions than from reaching the exact maximum of a single dynamical quantity. Our simulations demonstrate the former property across randomized networks; they do not establish that the biological architecture evolved to optimize information flux.

This interpretation agrees with earlier recurrent-network results showing that a strongly positive excitatory–inhibitory balance drives the dynamics into a fixed-point regime with minimal information flux, whereas sufficiently strong external perturbations can release the network from the attractor (Metzner and Krauss, 2022). In the present model, the large positive recurrent weights of the core provide precisely this dynamical substrate. Embedding inhibition, static biases, adaptive biases, and appropriately tuned noise differ in efficiency, but each can be understood as external regulation that moves the core away from its isolated fixed-point-like regime.

This distinction also explains why structural complexity can remain useful even when a simpler intervention performs better in a controlled simulation. The optimized biases were selected separately for each fixed core using an explicit information-flux objective or were learned under a prescribed activity target. In contrast, the embedding network generated heterogeneous negative mean inputs through its ordinary recurrent dynamics. It also supplied spatially correlated fluctuations and could, in a biological setting, respond to inputs and internal state changes that were absent from the static optimization experiment. These possible advantages motivate further tests of robustness and adaptation, but they should not be inferred from the present steady-condition comparisons alone.

The entropy decomposition provides an information-theoretic account of the different interventions. Mutual information between successive states obeys *I*(*Z*_*t*_; *Z*_*t*+1_) = *H*(*Z*_*t*+1_)−*H*(*Z*_*t*+1_|*Z*_*t*_). The isolated core lost a large amount of state entropy because it occupied a restricted, highly active part of state space. Its conditional entropy also decreased, indicating more predictable transitions, but this gain in predictability was insufficient to compensate for the loss of state diversity. The per-neuron matched means modestly reduced state entropy while reducing conditional entropy still more, thereby increasing mutual information. Optimized and adaptive biases instead increased both state entropy and transition uncertainty; their entropy gains were larger, leaving a positive net information-flux change.

These results clarify why bias regulation can be more effective than adding independent noise. Noise may help a trapped recurrent system explore additional states, as seen in the isolated-core noise sweep, but it can simultaneously make successive transitions less predictable. A suitable bias shifts the operating point without necessarily introducing an equally large stochastic contribution. Spatially correlated fluctuations nevertheless matter for reproducing the complete embedded result: once their instantaneous covariance was matched, Gaussian signals performed like the original embedding signals. The relevant distinction is therefore not simply “bias vs. noise”, but the balance between mean regulation, state-space exploration, transition uncertainty, and shared fluctuations.

For artificial recurrent systems, the results suggest that neuron-specific excitability regulation may provide a local alternative to exclusively global parameter tuning. The adaptive rule is particularly notable because it reached a high-flux regime using only each neuron's time-averaged activity and no global mutual-information calculation. However, the target *p*_*i*_ = 1/2 has a precise meaning only for the binary neurons considered here: it maximizes their marginal output entropy, but it neither guarantees maximal network mutual information nor establishes optimal performance on a computational task.

### 4.3 Relation to other works

The present study connects information-theoretic analysis of recurrent dynamics with research on core–periphery organization, neural homeostasis, noise-enhanced processing, and reservoir computing. The embedded-core architecture is related to core–periphery and rich-club structures observed across complex and neural networks (Colizza et al., 2006; Van Den Heuvel and Sporns, 2011; Sporns and Betzel, 2016). At the microcircuit level, the strong and non-random excitatory connections reported by Song et al. (2005) and Perin et al. (2011) provide experimental motivation for structured neuronal cores embedded within weaker background connectivity. Our results suggest one possible dynamical function for such an arrangement: the surrounding circuit can regulate the operating regime of a strongly recurrent subnetwork. They do not imply that this is the only function of core–periphery organization or that the same mechanism necessarily applies at larger anatomical scales.

The general principle that excitation–inhibition balance controls recurrent-network regimes is well established. Balanced excitatory and inhibitory activity can generate irregular, rapidly responsive dynamics (van Vreeswijk and Sompolinsky, 1996); varying excitation, inhibition, and external drive produces transitions among asynchronous, synchronous, and oscillatory states in sparse spiking networks (Brunel, 2000); and information capacity and transmission in cortical cultures and network models can peak at an intermediate excitation–inhibition ratio (Shew et al., 2011). These studies use dynamical systems and information measures that differ from the successive-state mutual information analyzed here. We therefore do not claim novelty for the stabilizing role of inhibition itself. The specific contribution of the present work is the paired reverse engineering of a strongly recurrent embedded core: separating neuron-specific temporal means, independent fluctuations, and spatial covariance; resolving the entropy and conditional-entropy contributions; comparing embedding with optimized and locally adaptive bias regulation; and validating the reduced information-flux indicator against the full core state.

The new weight-assignment controls make this connection more explicit. Concentrating strong excitatory weights within the core is a central Song-inspired feature of the ECM rather than an incidental parameter choice. The deterministic selection of the 30 largest excitatory weights is an idealized implementation of the experimentally observed heavy-tailed and non-random organization, not a literal reconstruction of a measured microcircuit. When this privileged strong-weight organization was removed, the embedding benefit disappeared because the resulting weakly recurrent core no longer occupied the same dynamical regime. This dependence therefore defines the mechanism's architectural boundary while reinforcing the role assigned here to the strong core.

The directional asymmetry revealed by the lesions is reminiscent of the distinction between driving and modulatory inputs in cortical circuits (Sherman and Guillery, 1998). Removing core outputs did not impair the embedding network's effect, whereas inhibitory input to the core strongly controlled its operating point. In this limited sense, the embedding signal acts more like a modulatory influence on excitability than like a content-bearing signal that must continuously participate in a closed core–embedding computation. This analogy should remain qualitative because the ECM does not reproduce the cellular and laminar mechanisms used to distinguish driving from modulatory pathways biologically.

The adaptive-bias rule is functionally related to homeostatic plasticity and regulation of intrinsic excitability, through which neurons stabilize their activity in the face of persistent changes (Turrigiano and Nelson, 2004; Turrigiano, 2008). It also shares the broader principle of activity-dependent local regulation with models such as the BCM rule (Bienenstock et al., 1982). What the present model adds is a direct link between such a local rule and a global measure of temporal dependence: regulating the marginal activity of each binary neuron placed the recurrent core in a high-flux regime. This is a model-specific result, not evidence that biological neurons generally target an on-probability of one half. Biological homeostatic targets depend on cell type, coding scheme, timescale, and energetic constraints.

The isolated-core noise sweep extends previous work on Recurrence Resonance, in which an intermediate noise level increases mutual information between successive states of a recurrent system (Krauss et al., 2019a; Metzner et al., 2024). The mechanism is related to stochastic resonance more generally, where noise can improve the response or information processing of nonlinear systems (Benzi et al., 1981; Wiesenfeld and Moss, 1995). Our results delimit this benefit: independent Gaussian noise partially rescued a core trapped by strong excitation, but remained much less effective than the embedded network or suitable biases. Moreover, the replacement controls revise the earlier interpretation of the embedding fluctuations. Their advantage over independent Gaussian input was reproduced by matching spatial covariance and therefore cannot be attributed specifically to non-Gaussianity.

Reservoir computing likewise emphasizes the importance of placing a recurrent network in a dynamical regime that supports memory and useful state representations (Jaeger, 2001; Büsing et al., 2010; Dambre et al., 2012; Schuecker et al., 2018). Common approaches regulate global properties such as recurrent gain, spectral scaling, input strength, or noise amplitude. The present results highlight a complementary possibility: heterogeneous neuron-specific biases can regulate the operating points of individual units, and a simple local rule can establish them without directly optimizing the recurrent weights. Whether this improves memory or task performance, rather than only successive-state mutual information, remains to be tested.

### 4.4 Open questions and outlook

The first major open question concerns the functional meaning of information flux. Mutual information between successive network states quantifies temporal statistical dependence, but high values do not by themselves imply efficient computation. A network can retain substantial information about its immediately preceding state while representing task-irrelevant dynamics, whereas useful computation may require selective forgetting, nonlinear transformation, or sensitivity to external input. Future studies should therefore vary bias and embedding conditions while measuring memory capacity, prediction, classification, or other explicit tasks. Such experiments would determine whether the high-flux regimes identified here are also computationally advantageous.

Relatedly, the term information flux should not be confused with causal or directed information transfer between particular neurons or populations. The pairwise and triplet mutual-information measures can reflect direct interactions, indirect pathways, common input, and collective state changes. Intervention-based lesions provide stronger mechanistic evidence for pathway necessity, but they do not reconstruct the full causal organization of the network. Future analyses could combine controlled perturbations with directed information-theoretic or causal-modeling approaches.

The triplet indicator was strongly validated against full-state mutual information for autonomous isolated cores across uniform-bias regimes, and alternative partitions produced consistent results. Nevertheless, this validation does not prove that the same quantitative relation holds for every architecture, time-dependent input, or manipulation. Semianalytical full-state calculations are not directly available for the complete embedded system because the core alone is then not autonomous. Longer empirical calculations, state-space models, or validation in additional tractable networks would further establish the range over which the reduced indicator remains reliable.

The Gaussian controls also leave a specific signal-processing question open. The spatial-covariance-matched control reproduced the instantaneous covariance of the embedding input but was not designed to reproduce its temporal autocovariance or power spectrum. The recorded embedding signals showed only weak lag-one correlations, but even weak temporal structure could affect recurrent dynamics over longer timescales. Gaussian controls matched to temporal covariance, followed by controls for higher-order dependencies, would permit a more complete hierarchy of signal features to be tested.

Several limitations follow from the abstraction level of the ECM. Its neurons are binary, stochastic, and synchronously updated; its synaptic weights are fixed; and only selected connectivity statistics were taken from layer 5 measurements. The model omits spikes, conduction delays, synaptic dynamics, cell-type diversity, plasticity, and ongoing sensory input. These simplifications made systematic reverse engineering and exact validation feasible, but they limit direct biological interpretation. Replication in asynchronous binary networks, spiking models, and data-constrained circuit reconstructions will be necessary to determine which mechanisms survive at greater biological realism.

A particularly important extension is to replace the deterministic top-weight core by probabilistic architectures that preserve the measured heavy-tailed weight distribution and conditional enrichment of strong or reciprocal connections. Likewise, inhibitory projections should ultimately be constrained by cell-type-specific anatomical and physiological data rather than represented by uniform population-level weights. Such models would test how broadly the present mechanism survives between the weakly recurrent assignment controls and the deliberately strong core analyzed here.

The adaptive experiment should likewise be extended beyond the single target *p*_*i*_ = 1/2. Other target activities, adaptation rates, observation timescales, and constraints on bias magnitude may produce different balances between entropy and predictability. It will also be important to perturb the core after adaptation, alter recurrent weights, or expose it to time-dependent inputs to test whether local activity regulation can maintain a high-flux regime under changing conditions rather than only reach one from a fixed initialization.

Finally, the analysis treated the peripheral and interneuron populations mainly as an embedding system that regulates the core. Their numerous weak connections may also process and store information, coordinate activity across multiple cores, or support robustness under external drive. Extending the information-theoretic analysis to the complete network and comparing different core–periphery architectures, network sizes, and excitation–inhibition structures could reveal when distributed embedding is preferable to direct bias regulation. This broader comparison would help separate properties specific to the present layer-5-inspired model from general principles of recurrent network organization.

## Data Availability

The complete data and analysis programs supporting the findings of this study are available in a public reproduction package at Dropbox: Embedded Core Model reproduction package (https://www.dropbox.com/scl/fo/t8zptocfc67mqs2mcyyn5/AAegbQ4wCvOcYWdSXEWN3EA?rlkey=cp4we8571zdo6n01loqpjmouh&dl=0). The package includes the generated network connectivity and weight matrices; simulation source code; parameter files and run configurations; raw and processed outputs underlying the 25-network ensemble analyses; the data and programs used for the full-state mutual-information and triplet-partition validation; all additional analyses reported in the Supplemental Material; and the numerical source data and plotting programs used to generate the figures and summary statistics. All internal paths are relative to the package root. This provisional Dropbox location will be replaced by a persistent repository identifier if one is assigned.

## Supplementary material

Data Sheet 1 Supplementary Methods, Results, Figures S1–S6 and Table S1 for the Embedded Core Model study.
