# Supplementary material for: Are cortical microcircuits optimized for information flux?—A simulation-based reverse engineering study
Source: Front Comput Neurosci. 2026 Oct 20;20:1886601. doi: 10.3389/fncom.2026.1886601 (PMC13727633; doi:10.3389/fncom.2026.1886601)
Supplement: Supplementary file 1 [file Data_Sheet_1.pdf]

1 Supplementary Methods

1.1 General design and statistical unit

All supplementary analyses used the same ensemble of 25 independently generated network realizations as the main manuscript. Comparisons between conditions were paired within a network whenever the topology was shared. Simulation replicates, trajectories, core-neuron pairs, and weight permutations were averaged or otherwise grouped within their parent network before ensemble inference. The network realization, therefore, remained the independent statistical unit throughout. Unless stated otherwise, uncertainty is reported as a two-sided 95% percentile-bootstrap confidence interval obtained by resampling the 25 network identifiers with replacement.

1.2 Entropy and conditional-entropy decomposition

For every central signal-control and bias condition, we decomposed the triplet mutual information according to

$$\Delta I = \Delta H - \Delta H_{\text{cond}}, \tag{1}$$

where all changes were calculated within the same network relative to the matching full embedding result. The signal-replacement conditions used the evaluation data underlying Fig. 6 of the main manuscript. Optimized uniform and per-neuron biases used the independent held-out evaluations underlying Fig. 7, and adaptive-bias values were first averaged across the two adaptation seeds and two held-out evaluation seeds within each network.

The analysis was performed separately for the intra-triplet and inter-triplet quantities defined in the main Methods. For graphical interpretation, ΔH and $-\Delta H_{\text{cond}}$ are shown as additive contributions, whose sum equals ΔI. Means and percentile-bootstrap 95% confidence intervals were calculated across the 25 paired network realizations using 20,000 resamples. The algebraic identity was verified for every network, condition, and triplet scope before aggregation. No additional simulations were required.

1.3 Adaptive intrinsic biases in the embedded ECM

The local bias-adaptation rule previously applied to the isolated core was repeated while the complete 125-neuron Embedded Core Model (ECM) remained connected and dynamically active. Only the ten intrinsic biases of the core neurons were updated,

$$b_i(t) = b_i(t-1) - \epsilon \left(z_i(t) - \frac{1}{2} \right), \tag{2}$$

with temperature $T = 5$, learning rate $\epsilon = 0.01$, and 100,000 adaptation updates. Two independent adaptation seeds were used for each network. The learned vector was defined by averaging each bias over the final 20,000 updates. Each learned vector was evaluated on two independent held-out trajectories with 5,000 burn-in updates and 50,000 recorded transitions. Adaptation and evaluation replicates were averaged within network before comparison with the embedded baseline and the previously evaluated isolated-core baseline, optimized-bias, and adaptive-bias conditions. Confidence intervals used 20,000 paired network bootstrap resamples.

1.4 Independent inhibitory target-strength grid

To test whether the embedded operating regime improved when inhibition of the strongly coupled core and weakly coupled periphery could differ, the values of existing inhibitory edges were varied independently without changing the connectivity pattern. The tested interneuron-to-core weights were -10, -8, -6, -5, -4, -2, and 0. The tested interneuron-to-periphery weights were -100,

-60, -40, -30, -20, -15, -12, -10, -8, -6, -5, -4, -2, and 0. The extended periphery range was included because an initial grid placed multiple optima at its lower boundary.

Candidate values were assessed using two training trajectories per grid point and network, each with 1,000 burn-in updates and 10,000 recorded transitions. We selected both the single weight pair maximizing the ensemble-mean training indicator and the pair maximizing the training indicator within each network. Independent held-out trajectories with 5,000 burn-in updates and 50,000 recorded transitions then evaluated the baseline ECM ($w_{I\to C} = w_{I\to P} = -5$), the common optimum, and the network-specific optimum. Selection and evaluation trajectories used distinct random seeds. The held-out paired effects were summarized across networks using 20,000 bootstrap resamples.

1.5 Input consequences of pathway lesions

The intact ECM and seven directional or combined lesions were evaluated for every network: core-to-periphery, core-to-interneurons, interneurons-to-core, periphery-to-core, periphery-to-interneurons, interneurons-to-periphery, and all core-to-embedding projections. Each condition used a 5,000-update burn-in followed by 50,000 recorded transitions at $T = 5$, with a common dynamics seed to preserve paired comparisons.

In addition to the triplet information measures, we recorded mean activity in all three populations and decomposed the input received by each core neuron into recurrent core, direct peripheral, and direct inhibitory components. For each input component and their sums, we retained neuron-specific temporal means and variances and the complete instantaneous 10×10 covariance matrix. Lesion effects were defined as lesion minus intact ECM. The principal mechanistic comparisons were the periphery-to-interneuron and interneuron-to-periphery lesions; all seven lesions were retained to show their magnitude relative to direct pathways. Confidence intervals used 20,000 paired resamples of network identifiers.

1.6 Projection overlap and spatial input covariance

For each of the 45 unordered pairs of core neurons in every network, presynaptic-source overlap was calculated separately for the excitatory periphery and inhibitory interneuron population. Binary overlap was summarized by the shared-source count, Jaccard index, and binary cosine similarity; a weighted cosine similarity additionally incorporated synaptic weights. These structural quantities were related to the component-specific instantaneous input covariance and correlation obtained from the intact trajectories of the lesion analysis.

The primary association was estimated within networks, asking whether core-neuron pairs with more shared presynaptic sources also received more strongly covarying input in the same realized circuit. Pooled within-network associations used cluster-bootstrap intervals that resampled whole networks, and within-network permutation tests shuffled the structural-overlap values among pairs separately in each network. We additionally summarized the 25 within-network Spearman correlations and compared network-mean overlap with network-mean covariance. Twenty thousand bootstrap and 10,000 permutation resamples were used. Core-neuron pairs were never treated as independent experimental units.

1.7 Core activity along the isolated-core noise sweep

The existing isolated-core Gaussian-noise sweep was reanalyzed at noise standard deviations $\sigma \in \{0, 0.5, 1, 1.5, 2, 2.5, 3, 4, 6, 8, 10\}$. For each noise level and network, the stored 50,000-transition trajectory provided the flux indicator, intra-triplet and inter-triplet mutual information, successor-state entropy, conditional entropy, and mean core on-probability. Ensemble summaries and comparisons with $\sigma = 0$ were paired by network and used 20,000 bootstrap resamples. We also determined the

maximizing noise level separately within each network and calculated the network-centered association between activity and flux on the rising branch from $\sigma = 0$ to $\sigma = 2$.

1.8 Robustness to excitatory weight assignment

The baseline construction assigns the 30 largest sampled excitatory weights to the 30 core-to-core edges. Two controls tested sensitivity to this ranked assignment while holding the complete excitatory weight multiset, excitatory edge locations, and all inhibitory connections fixed. In the global-random control, all positive weights were randomly permuted across the existing excitatory edges. In the targeted-swap control, only the 30 core weights were exchanged with 30 randomly selected noncore weights, leaving every other edge weight unchanged. Five independent global permutations and five targeted swaps were generated per network.

Embedded and isolated dynamics were evaluated after 5,000 burn-in updates and for 50,000 recorded transitions, using the same dynamics seed for paired conditions. Control replicates were averaged within network before ensemble inference. We compared the core-weight distribution, absolute information-flux indicators, embedding gain (embedded minus isolated), entropy components, and mean core activity. Twenty thousand bootstrap resamples of networks were used. These manipulations are architectural sensitivity controls and are not intended as alternative biological null models.

1.9 Statistical sensitivity analyses

We assessed whether conclusions based on 25 network realizations depended on the percentile bootstrap. Twenty prespecified paired effects from the adaptation, inhibitory-grid, lesion, noise, and weight-assignment analyses were examined using 50,000 percentile-bootstrap intervals, bias-corrected and accelerated (BCa) intervals, paired-network Student t intervals with 24 degrees of freedom, paired sign-flip tests with 200,000 random permutations, exact two-sided sign tests, standardized paired effects, and leave-one-network-out means. Resampling always retained the pairing of conditions within a network. The procedures are treated as converging sensitivity analyses of the same effects, not as independent hypothesis tests.

2 Supplementary Results

2.1 Bias and noise affect both terms of the mutual information

Isolation caused a large loss of successor-state entropy relative to full embedding, $\Delta H = -1.164$ bit for both triplet scopes (Fig. S1). This loss was largely offset by a decrease in conditional entropy, corresponding to contributions of $-\Delta H_{\text{cond}} = +1.126$ bit for the intra-triplet quantity and $+1.117$ bit for the inter-triplet quantity. The incomplete cancellation nevertheless left substantial reductions of 38.10×10^{-3} bit and 47.15×10^{-3} bit, respectively. Thus, the isolated core becomes more predictable, but this does not compensate for its greatly restricted state occupation. Empirical replay changed neither component appreciably and therefore reproduced full embedding.

Replacement by the per-neuron matched means reduced the successor-state entropy by 82.16×10^{-3} bit, but reduced conditional entropy still more strongly. The resulting net increases were 6.96×10^{-3} bit for the intra-triplet measure and 6.57×10^{-3} bit for the inter-triplet measure. Independent Gaussian input produced only small component changes, but their balance was unfavorable, yielding decreases of 1.92×10^{-3} bit and 3.15×10^{-3} bit. For the spatial-covariance-matched Gaussian control, small entropy and conditional-entropy changes nearly cancelled, leaving differences from full embedding whose confidence intervals included zero.

The optimized and adaptive bias conditions followed a distinct pattern. Relative to full embedding, they increased successor-state entropy by approximately 0.296–0.432 bit. Their conditional entropies

also increased, producing negative contributions of approximately -0.250 to -0.391 bit, but these increases were smaller than the entropy gains. The remaining positive differences ranged from 30.06×10^{-3} to 40.81×10^{-3} bit for the intra-triplet measure and from 45.50×10^{-3} to 56.92×10^{-3} bit for the inter-triplet measure. The adaptive-bias condition produced the largest mean entropy gain and the largest net information-flux increase of the tested conditions.

The decomposition therefore does not support a strict separation in which biases act only through entropy while noise acts only through conditional entropy. Both terms generally change together. The information-flux differences arise from their balance: isolation strongly reduces both entropies, matched means improve predictability more than they reduce state diversity, and optimized or adaptive biases increase state diversity more than they increase transition uncertainty.

2.2 Bias adaptation improves the embedded ECM but does not close the performance gap

Adaptive intrinsic biases increased the embedded flux indicator from 116.07 to 177.04 (Fig. S2a). The paired increase was 60.97 (95% CI 46.44 to 76.30) and was positive in all 25 networks. Adaptation increased successor-state entropy more than conditional entropy, so both intra-triplet and inter-triplet mutual information increased (Fig. S2b). The mean learned intrinsic bias was -0.742. Mean tail activity reached 0.49993, and the largest absolute deviation of any neuron-level adaptation-run mean from the target of 0.5 was 0.00460 (Fig. S2c).

The adapted embedded ECM nevertheless remained below the adapted isolated core by 36.76 indicator units (95% CI 31.28 to 42.82 in favor of the isolated condition). Its mean also remained below the isolated core with optimized uniform biases (191.64), optimized neuron-specific biases (208.82), or adaptive biases (213.79). Thus, the baseline ECM is not limited only by the absence of homeostatic bias regulation: adding that regulation substantially improves the embedded network, but the tested embedding still does not maximize the information-flux measure.

2.3 Unequal inhibitory targeting improves the ECM

The common training-grid optimum retained the baseline interneuron-to-core weight of -5 but strengthened interneuron-to-periphery projections to -30 (Fig. S3a). On independent held-out trajectories, the common optimum increased the mean indicator from 116.07 to 122.97. The paired improvement was 6.90 (95% CI 4.84 to 8.98) and was positive in 22 of 25 networks (Fig. S3c). The training profile was effectively flat under still stronger periphery inhibition: ensemble training indicators were 128.82, 128.78, 128.78, and 128.78 at weights -30, -40, -60, and -100, respectively. The selected common value therefore lies at the beginning of a strong-inhibition plateau rather than representing a sharply localized biological estimate.

Network-specific selection produced a larger held-out mean of 129.94, corresponding to an improvement of 13.87 (95% CI 10.68 to 17.12; positive in 25/25 networks). Selected core-target weights were concentrated between -8 and -4, whereas selected periphery-target weights were widely distributed across the extended grid (Fig. S3b). These findings answer the concern about identical inhibitory weights directly: allowing unequal strengths improves the ECM, especially through stronger control of the excitatory periphery, but does not approach the adaptive-bias result and does not establish a unique biologically matched parameterization.

2.4 Mechanisms of indirect pathway lesions

Removing periphery-to-interneuron projections reduced the flux indicator by 12.38 (95% CI -18.12 to -6.84), with intra-triplet and inter-triplet mutual-information changes of -6.16 millibit (95% CI -9.20 to -3.36) and -6.22 millibit (95% CI -9.55 to -2.87), respectively (Fig. S4a). Interneuron

activity fell by 0.0875, making inhibitory core input 1.191 units less negative, while increased peripheral activity added 0.363 units of excitation. Total mean embedding input consequently rose by 1.554 (95% CI 1.457 to 1.651), and mean core activity rose by 0.0912 (95% CI 0.0831 to 0.1001; Fig. S4b,c). Input variance and absolute off-diagonal covariance increased rather than decreased. The information loss is therefore not explained by removal of fluctuations; it accompanies a mean shift toward the high-activity regime.

Removing interneuron-to-periphery projections reduced the indicator by 25.21 (95% CI −29.46 to −20.77), with intra-triplet and inter-triplet changes of −11.76 millibit (95% CI −14.50 to −9.00) and −13.46 millibit (95% CI −16.22 to −10.70). Disinhibition increased peripheral excitation of the core by 3.281 units and also recruited interneurons, making their direct core input 1.845 units more negative. The net embedding mean still rose by 1.435 (95% CI 1.225 to 1.629), and core activity rose by 0.0810. In this lesion, embedding-input variance and covariance fell substantially. Across networks, the flux-indicator change correlated with the change in mean embedding input ($r = -0.642$), whereas the association with variance change was weaker ($r = -0.542$).

Both indirect lesions therefore perturb the mean operating point; loss of shared fluctuations contributes additionally to the interneuron-to-periphery effect. Their effects are appreciably smaller than direct removal of interneuron-to-core input, which changed the indicator by −99.07. We consequently interpret the indirect pathways as modulatory components of the embedded operating regime, not as its dominant cause or as the sole link to cortical connectivity data.

2.5 Shared projections predict instantaneous input covariance

The projection-overlap analysis contained 45 core-neuron pairs in each of 25 networks, yielding 1,125 pairs for each embedding source population. Core-neuron pairs shared on average 1.137 excitatory-periphery sources (mean Jaccard index 0.0578). Within networks, greater Jaccard overlap predicted larger input covariance: pooled $r = 0.522$ (cluster-bootstrap 95% CI 0.461 to 0.578; permutation $p < 10^{-4}$). The mean within-network Spearman correlation was 0.617 (95% CI 0.579 to 0.656) and was positive in every network (Fig. S4d,f).

Inhibitory projections had a mean shared-source count of 0.305 and mean Jaccard index of 0.0618. Their association with input covariance was stronger: pooled within-network $r = 0.857$ (95% CI 0.794 to 0.924; permutation $p < 10^{-4}$), with a mean within-network Spearman correlation of 0.727 (95% CI 0.695 to 0.757; positive in 25/25 networks; Fig. S4e,f). Associations also remained positive for input correlation and for weighted-overlap measures. Projection overlap is therefore a robust structural source of shared core input in this model, particularly for uniform-weight inhibitory edges. It is not a complete explanation because input covariance also depends on presynaptic activity, correlations among distinct sources, and synaptic weights.

2.6 Moderate noise moves the isolated core away from saturation

The isolated-core flux indicator followed a nonmonotonic noise dependence and reached its ensemble maximum at $\sigma = 2$: 41.58 (95% CI 38.45 to 44.86; Fig. S5a). Relative to zero noise, the paired increase was 10.70 (95% CI 7.64 to 13.74) and was positive in 22 of 25 networks. Mean core activity simultaneously fell from 0.8815 (95% CI 0.8727 to 0.8906) at $\sigma = 0$ to 0.7731 (95% CI 0.7628 to 0.7833) at $\sigma = 2$, a paired change of −0.1084 (95% CI −0.1134 to −0.1034; lower in 25/25 networks; Fig. S5b). On the rising branch, the network-centered activity-flux association was $r = -0.734$.

At $\sigma = 2$, intra-triplet successor-state entropy increased by 0.8019 bit and conditional entropy by 0.7975 bit, leaving a smaller positive mutual-information difference. Individual-network optima occurred at $\sigma = 1$ (5 networks), 1.5 (7), 2 (9), 2.5 (3), or 3 (1); every network had an optimum above zero noise. The maximum thus coincides with movement away from the near-saturated high-activity regime predicted by the single-neuron analysis. Noise is not beneficial without limit: beyond the intermediate optimum, transition uncertainty continues to grow and temporal dependence declines.

2.7 The embedding effect depends on the strong recurrent-weight skeleton

The ranked baseline assigned all 30 globally largest excitatory weights to the core, giving a mean core-edge weight of 4.762. Global randomization reduced this mean to 0.767, and the targeted core-weight swap reduced it to 0.670 (Fig. S5c). Under ranked assignment, embedded and isolated indicators were 115.96 and 30.88, respectively, yielding an embedding gain of 85.08 (95% CI 73.16 to 98.81; positive in 25/25 networks; Fig. S5d,e).

After global random assignment, embedded and isolated means were 8.14 and 11.94, producing a gain of −3.80 (95% CI −4.34 to −3.27; positive in 0/25 networks). The targeted swap gave corresponding means of 5.41 and 8.22 and a gain of −2.81 (95% CI −3.28 to −2.37; positive in 0/25 networks). The targeted result shows that reversal of the embedding effect is not an artifact of permuting all noncore excitatory weights. Embedded core activity changed from 0.5477 under ranked assignment to 0.3104 and 0.3069 in the two controls (Fig. S5f).

These controls identify the strong recurrent-weight skeleton as a necessary modeling ingredient for the reported baseline embedding advantage. They do not show that concentrated strong weights are incompatible with experimental observations of nonrandom strong excitatory subnetworks. Rather, the deterministic top-30 assignment is an idealized, Song-motivated construction whose exact strength and allocation are not fixed by those data. With strong recurrence, the isolated core tends toward the low-flux fixed-point regime and benefits from external regulation; with ordinary weak core weights, that dynamical problem and the corresponding embedding advantage disappear.

2.8 Conclusions are stable across alternative paired-network analyses

Percentile-bootstrap, BCa, and paired-network t intervals gave concordant conclusions for the principal effects (Fig. S6a). For example, the embedded-minus-isolated baseline difference was 85.45, with respective intervals of 73.26 to 99.14, 74.59 to 101.23, and 71.50 to 99.40. The embedded-adaptation gain was 60.97, with intervals of 46.80 to 76.32, 47.72 to 77.55, and 45.03 to 76.90. Common inhibitory tuning, network-specific tuning, and the $\sigma = 2$ noise effect likewise retained positive intervals under all three methods. Ranked-weight embedding gain remained positive, whereas both alternative weight-assignment gains remained negative.

The smaller periphery-to-interneuron and interneuron-to-periphery lesion effects were also directionally stable (Fig. S6b,c). For the periphery-to-interneuron lesion, the intra-triplet change was −6.16 millibit, with percentile, BCa, and paired-t intervals of −9.21 to −3.37, −9.49 to −3.58, and −9.31 to −3.01, respectively; 21 of 25 paired effects were negative. The corresponding inter-triplet effect was negative in 19 of 25 networks. Both interneuron-to-periphery components were negative in all 25 networks. Leave-one-network-out estimates showed no single realization driving these results (Fig. S6d).

Across all 20 prespecified effects, the three interval methods agreed on a nonzero direction for 16. Effect magnitude, sign consistency, and leave-one-out stability are emphasized rather than treating exclusion of zero as a binary significance decision. The sensitivity analyses converge on the same conclusions while making the limitations of inference from 25 independent networks explicit.

3 Supplementary Figures

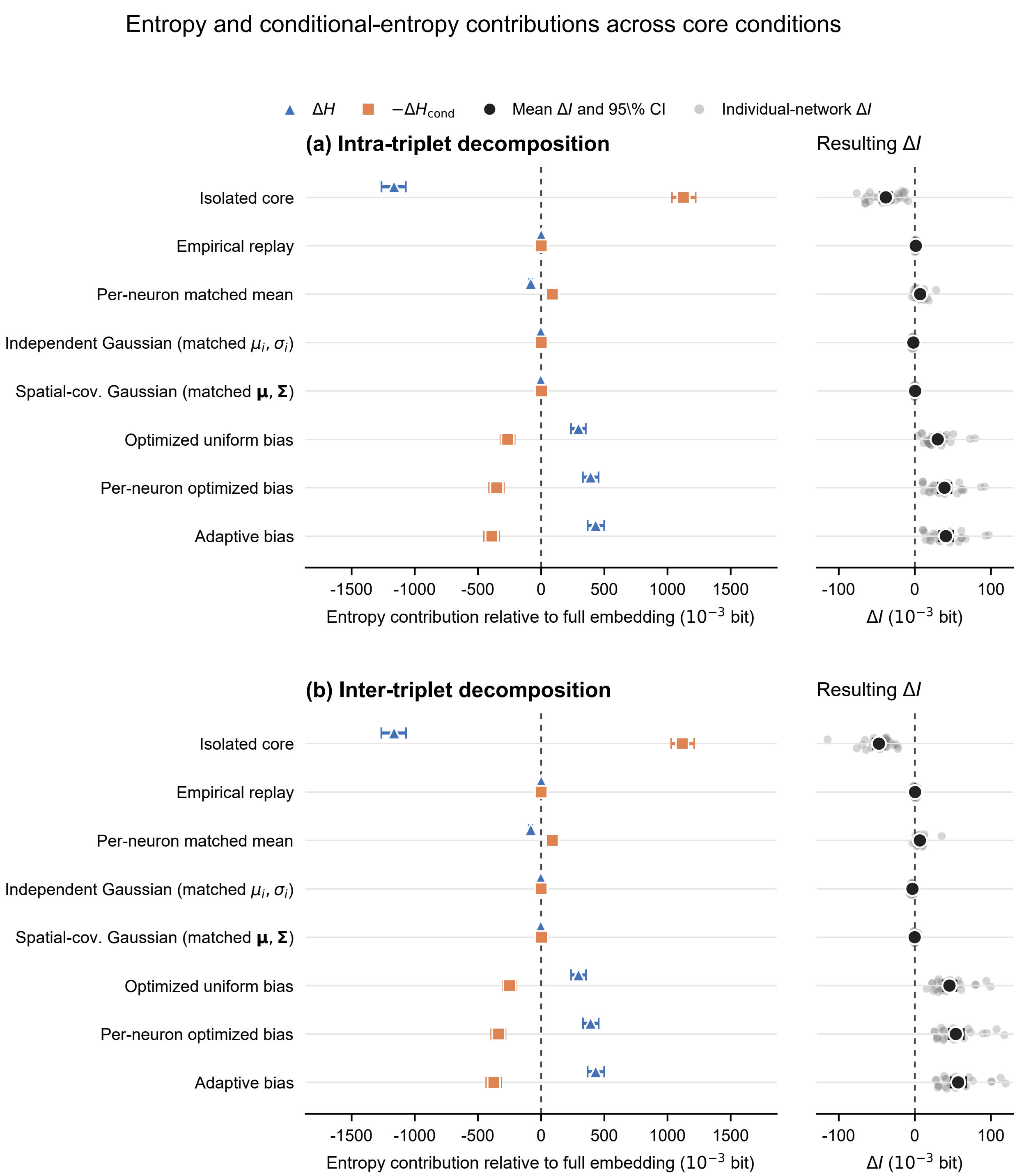

Figure S1: Entropy and conditional-entropy decomposition across 25 independently generated networks. Changes are paired within networks and expressed relative to the corresponding full-embedding evaluation. (a) Intra-triplet quantities. (b) Inter-triplet quantities. Blue triangles show the change in successor-state entropy ΔH; orange squares show the additive conditional-entropy contribution $-\Delta H_{\mathrm{cond}}$. Their sum is the mutual-information change ΔI, shown on the expanded axes at right. Large black circles and whiskers denote ensemble means and percentile-bootstrap 95% confidence intervals; small gray circles show the 25 individual-network values of ΔI. The signal-replacement conditions correspond to Fig. 6 of the main manuscript, whereas the optimized and adaptive conditions correspond to Fig. 7. Adaptive quantities were averaged over the two adaptation seeds and two held-out evaluation seeds within each network.

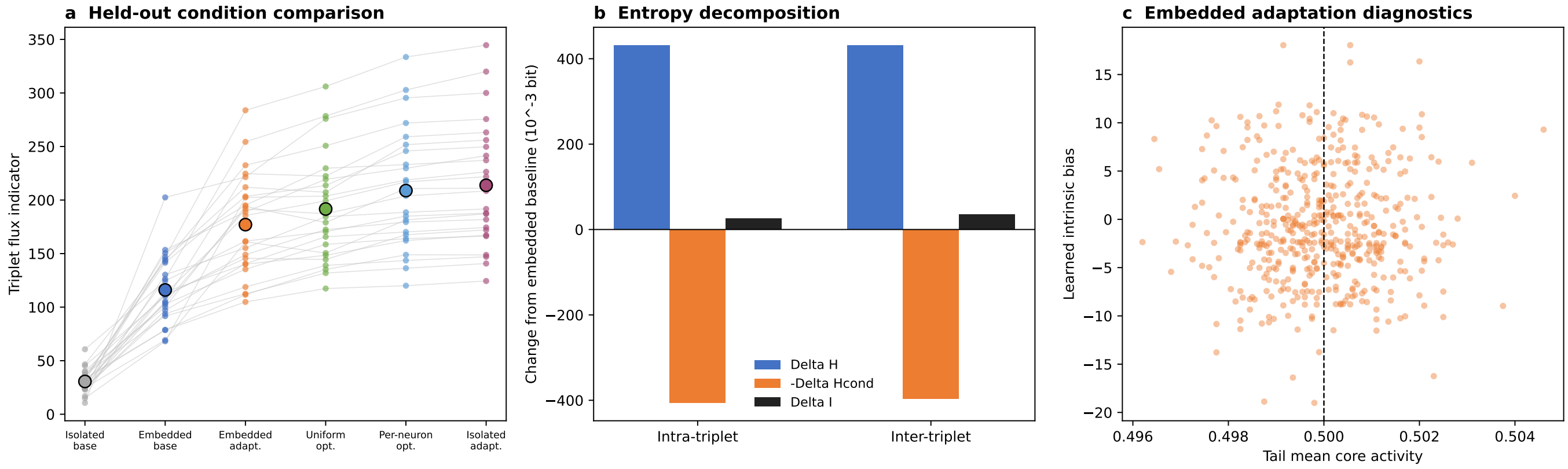

Figure S2: Adaptive intrinsic biases in the fully embedded ECM. The complete 125-neuron network remained connected during adaptation, and only the ten intrinsic biases of the core neurons were updated toward a target on-probability of 0.5. (a) Held-out flux-indicator values for the isolated and embedded baselines, the adaptively regulated embedded ECM, and the previously evaluated optimized and adaptive isolated-core conditions. Small points show the 25 individual networks; large symbols and whiskers show ensemble means and paired percentile-bootstrap 95% confidence intervals. Embedded adaptation increased the indicator by 60.97 (95% CI 46.44 to 76.30) relative to the embedded baseline, with positive changes in 25/25 networks, but remained below adaptive regulation of the isolated core. (b) Changes in successor-state entropy, the additive conditional-entropy contribution, and mutual information relative to the embedded baseline. (c) Learned intrinsic biases plotted against the tail mean activity of each core neuron. Adaptation reached a mean tail activity of 0.49993; the maximum absolute neuron-level deviation from 0.5 was 0.00460.

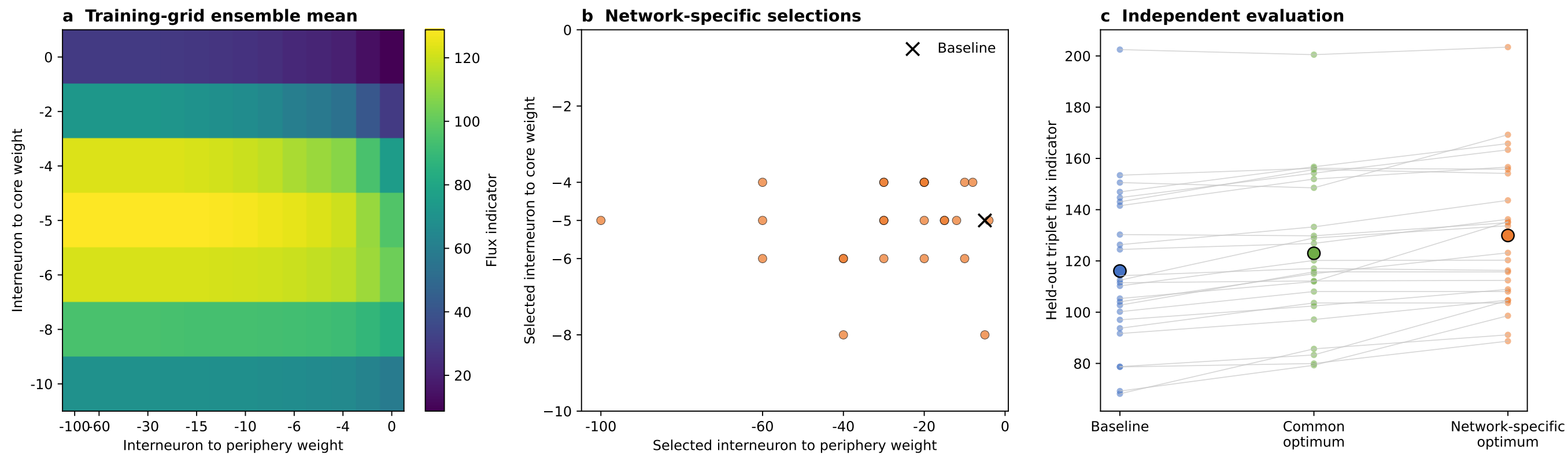

Figure S3: Independent tuning of inhibitory projection strengths to core and periphery. (a) Ensemble-mean flux indicator on the two-dimensional training grid of interneuron-to-core and interneuron-to-periphery weights. (b) Network-specific settings selected on the training trajectories; the baseline common inhibitory weight is indicated for reference. (c) Independent held-out evaluations of the baseline, the common grid optimum, and the network-specific optima. The common optimum ($w_{I\to C} = -5$, $w_{I\to P} = -30$) increased the indicator by 6.90 (95% CI 4.84 to 8.98; 22/25 positive networks), whereas network-specific selection increased it by 13.87 (95% CI 10.68 to 17.12; 25/25 positive networks). Thus, allowing unequal inhibitory targeting improves the embedded operating regime without making the baseline ECM globally optimal.

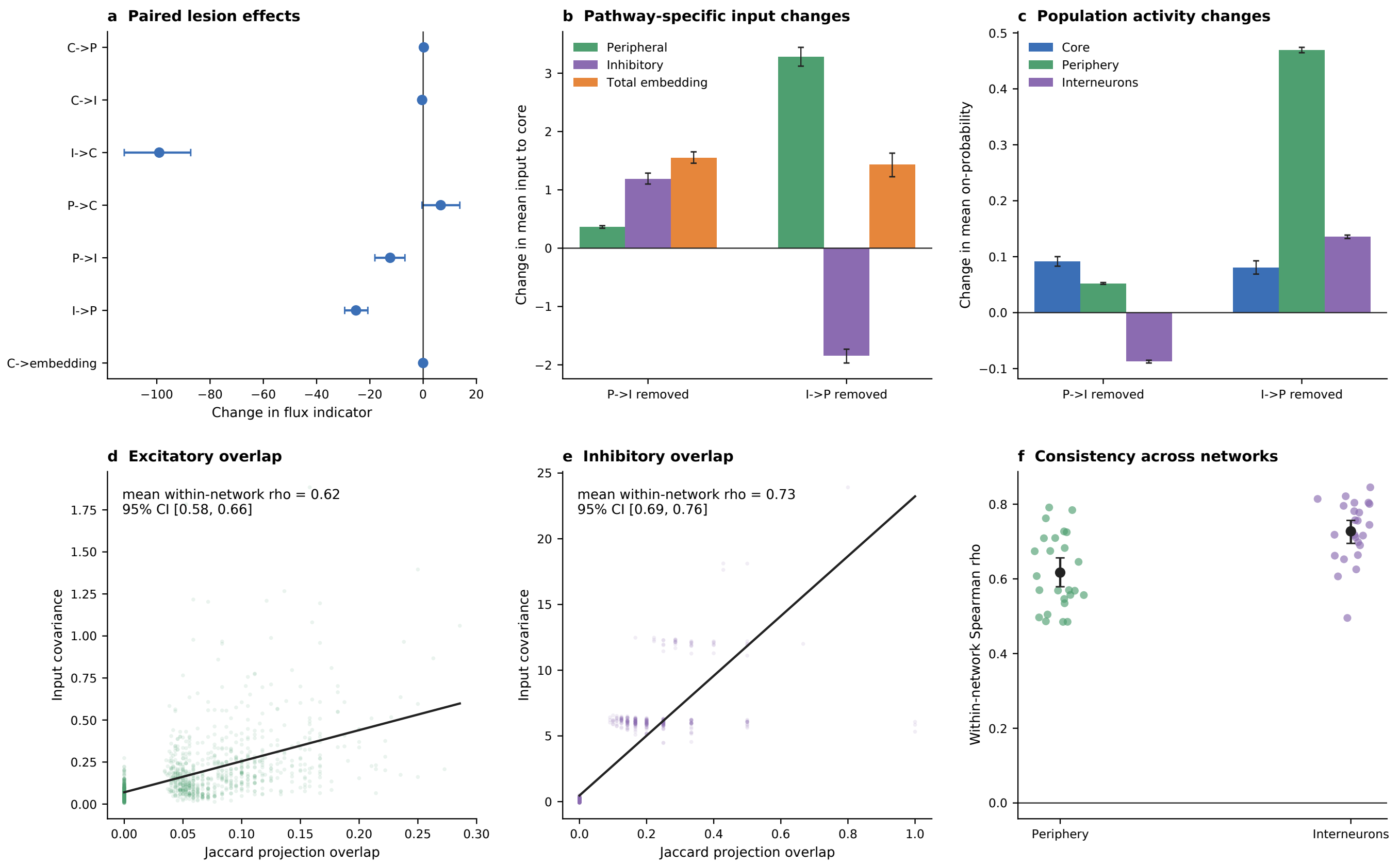

Figure S4: Mechanisms of lesion effects and structural origin of shared embedding input. All lesion differences are lesion minus intact ECM and are paired across 25 networks. (a) Changes in the flux indicator for all directed pathway lesions. (b) Changes in the mean peripheral, inhibitory, and total embedding input received by core neurons after removal of the periphery-to-interneuron ($P \to I$) or interneuron-to-periphery ($I \to P$) pathway. (c) Corresponding activity changes in the core, periphery, and interneuron populations. Both indirect lesions shift the core toward higher activity; the $I \to P$ lesion additionally reduces embedding-input variance and covariance. (d,e) Pairwise Jaccard overlap of presynaptic projections versus instantaneous input covariance for excitatory-periphery and inhibitory-interneuron sources. Points are core-neuron pairs pooled for display; lines are descriptive linear fits. (f) Within-network Spearman associations for all 25 networks, with ensemble means and network-bootstrap 95% confidence intervals. Projection overlap predicts covariance consistently for both source populations, particularly for the uniform-weight inhibitory projections, but is not asserted to be the sole source of covariance.

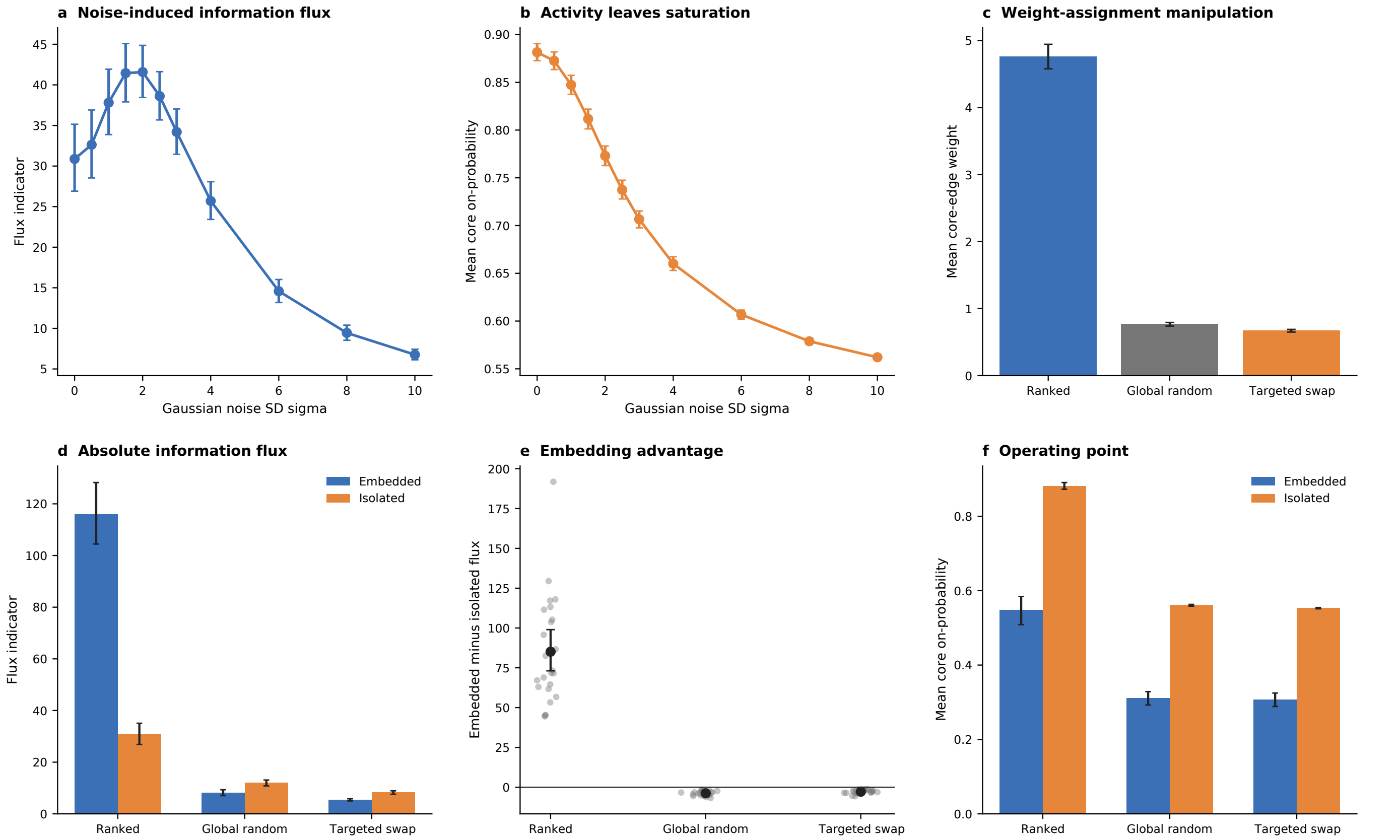

Figure S5: Dynamical robustness to external noise and excitatory weight assignment. (a) Flux indicator and (b) mean core on-probability along the isolated-core Gaussian-noise sweep. The ensemble indicator peaks at $\sigma = 2$, where mean activity has moved reproducibly away from the near-saturated state; larger noise subsequently increases transition uncertainty and reduces temporal dependence. (c) Mean core-edge weights under ranked assignment, global random assignment, and targeted exchange of only the 30 core weights with 30 noncore weights. (d) Absolute embedded and isolated flux indicators, (e) paired embedding advantages, and (f) corresponding mean core activities under the three assignments. Gray points in panel e show individual-network gains; black symbols and whiskers show ensemble means and network-bootstrap 95% confidence intervals. The ranked condition yielded a positive embedding gain of 85.08 (95% CI 73.16 to 98.81; 25/25 positive), whereas global randomization and targeted swapping yielded gains of -3.80 and -2.81, respectively (0/25 positive for both). These are architectural sensitivity controls: they demonstrate dependence on the strong recurrent-weight skeleton, not that the alternative assignments constitute biological null models.

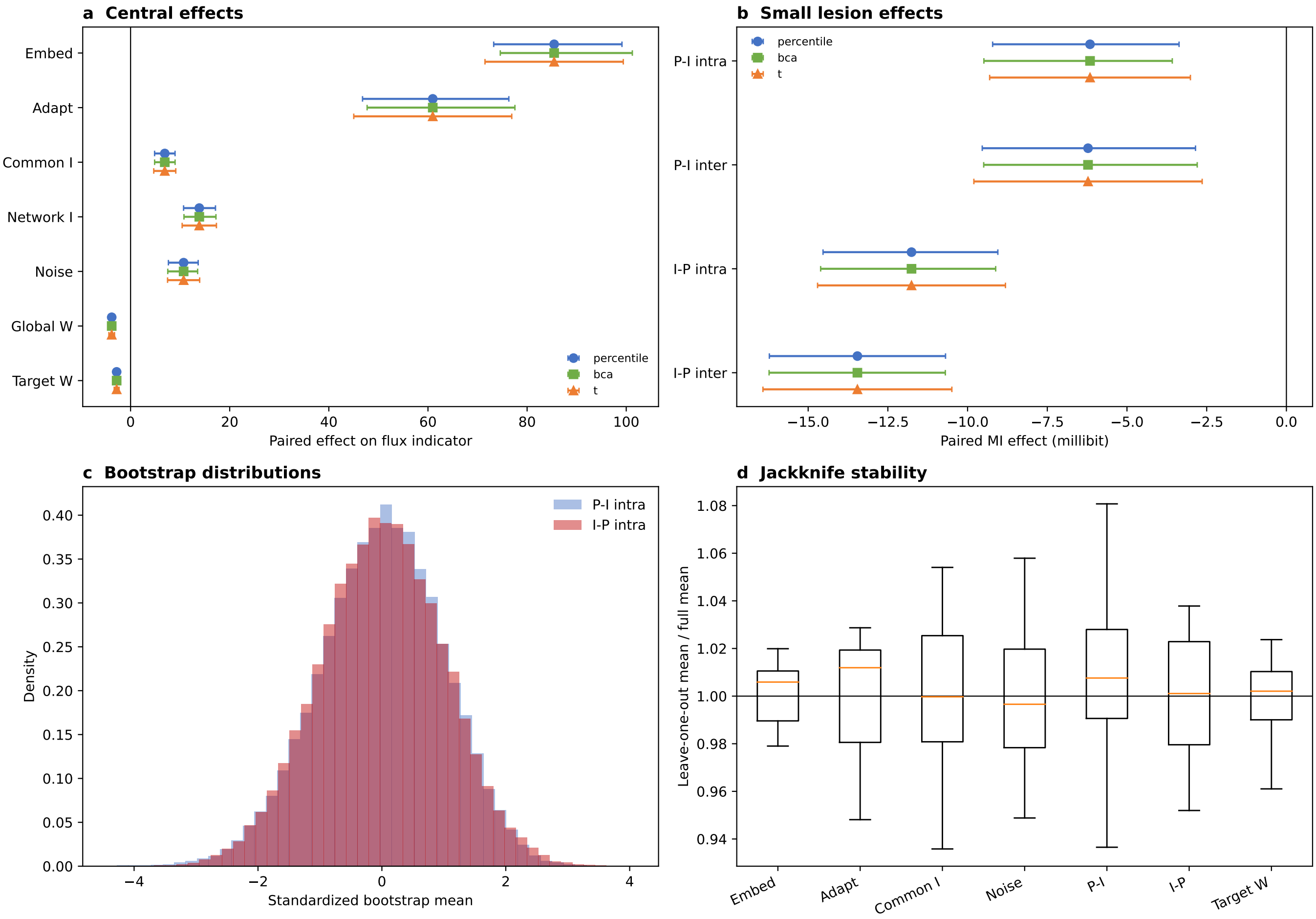

Figure S6: Statistical sensitivity across 25 paired network realizations. Networks, rather than trajectories, are the independent statistical units. (a) Central paired effects and (b) the smaller periphery-to-interneuron and interneuron-to-periphery lesion effects, shown with percentile-bootstrap, bias-corrected and accelerated (BCa), and paired-network t intervals. (c) Bootstrap distributions for the small lesion effects. (d) Leave-one-network-out means normalized by the corresponding full-sample mean. Paired sign-flip tests, exact sign counts, and complete numerical results are provided in the accompanying source tables. All three interval methods agree on a nonzero direction for 16 of 20 prespecified effects. These analyses are converging robustness checks and are not treated as independent hypothesis tests.

4 Supplementary Tables

Table S1: Paired entropy decomposition relative to full embedding. Entries are ensemble means with bootstrap 95% confidence intervals in units of 10^{-3} bit. The identity $\Delta I = \Delta H + (-\Delta H_{\text{cond}})$ holds before rounding.

Condition	ΔH	$-\Delta H_{\text{cond}}$	ΔI
Intra-triplet decomposition			
Isolated core	−1164.16 [−1263.89, −1071.26]	1126.06 [1034.85, 1222.80]	−38.10 [−46.06, −30.45]
Empirical replay	0.41 [−1.17, 2.11]	0.83 [−0.89, 2.54]	1.24 [0.86, 1.63]
Per-neuron matched mean	−82.16 [−95.72, −69.55]	89.12 [76.89, 102.23]	6.96 [4.35, 9.85]
Independent Gaussian	−2.82 [−4.68, −0.98]	0.90 [−1.05, 2.89]	−1.92 [−2.55, −1.33]
Spatial-cov. Gaussian	−3.13 [−4.52, −1.83]	3.50 [2.03, 5.06]	0.37 [−0.07, 0.83]
Optimized uniform bias	295.54 [236.39, 352.95]	−265.48 [−321.86, −209.82]	30.06 [23.28, 37.42]
Per-neuron optimized bias	391.46 [330.24, 454.25]	−352.55 [−411.89, −295.76]	38.92 [30.44, 47.92]
Adaptive bias	431.54 [368.42, 498.84]	−390.74 [−454.82, −332.42]	40.81 [32.06, 50.03]
Inter-triplet decomposition			
Isolated core	−1164.16 [−1264.77, −1069.58]	1117.01 [1029.30, 1211.10]	−47.15 [−55.37, −40.04]
Empirical replay	0.41 [−1.19, 2.14]	−0.11 [−1.90, 1.63]	0.30 [−0.24, 0.86]
Per-neuron matched mean	−82.16 [−95.67, −69.48]	88.73 [76.66, 101.82]	6.57 [4.23, 9.63]
Independent Gaussian	−2.82 [−4.68, −1.02]	−0.33 [−2.19, 1.58]	−3.15 [−3.80, −2.51]
Spatial-cov. Gaussian	−3.13 [−4.58, −1.85]	2.89 [1.48, 4.41]	−0.25 [−0.81, 0.39]
Optimized uniform bias	295.54 [237.71, 354.23]	−250.04 [−303.20, −196.25]	45.50 [37.48, 54.59]
Per-neuron optimized bias	391.46 [330.88, 455.07]	−337.63 [−395.41, −282.37]	53.83 [44.64, 64.17]
Adaptive bias	431.54 [367.99, 498.52]	−374.63 [−434.40, −316.86]	56.92 [47.36, 67.58]
